# Fe–AC nanocomposites derived from agro-waste biosorbents: a systematic review of synthesis routes, adsorption mechanisms, regeneration, and environmental sustainability

**DOI:** 10.1039/d6ra07855a

**Published:** 2026-10-05

**Authors:** Bijoylakshmi Boruah, Pratap Kumar Swain, Manash Ranjan Das

**Affiliations:** a NIT Arunachal Pradesh India 791113 pratapphy200@gmail.com; b CSIR-North East Institute of Science and Technology Jorhat Assam India 785006

## Abstract

Heavy metals, synthetic dyes, pharmaceuticals, pesticides, and other persistent contaminants continue to challenge water-treatment systems and require adsorbents that combine high affinity, recoverability, and practical reusability. Iron oxide-grafted agro-waste-derived activated carbon (Fe–AC) composites integrate the porosity and surface chemistry of biomass-derived carbon with Fe_2_O_3_/Fe_3_O_4_-based binding, redox activity, and magnetic separation. This systematic review evaluates synthesis routes, physicochemical characterization, contaminant-specific adsorption mechanisms, regeneration behavior, and the available evidence on environmental and economic implications. Particular attention is given to differences between inorganic and organic pollutant removal, the molecular-level roles of Fe–OH/Fe–O sites and carbon–surface interactions, and the influence of pH, competing ions, natural organic matter, and experimental conditions on reported adsorption performance. The literature indicates strong laboratory-scale potential, but direct cross-study ranking of adsorption capacities is limited by heterogeneous test conditions. Major barriers to practical translation include iron leaching, loss of capacity during regeneration, matrix interference, limited continuous-flow and real-wastewater validation, and the absence of harmonized quantitative life-cycle and techno-economic assessments. These limitations define priorities for standardized benchmarking, mechanistic validation, long-term cycling, pilot-scale testing, and environmentally robust Fe–AC design.

## Abstract 2

Agro-waste-derived Fe–AC couples porous carbon adsorption with iron-oxide binding and magnetic recovery. Performance depends on water chemistry, while iron leaching, regeneration losses and limited pilot-scale evidence remain key barriers.

## Graphical abstract

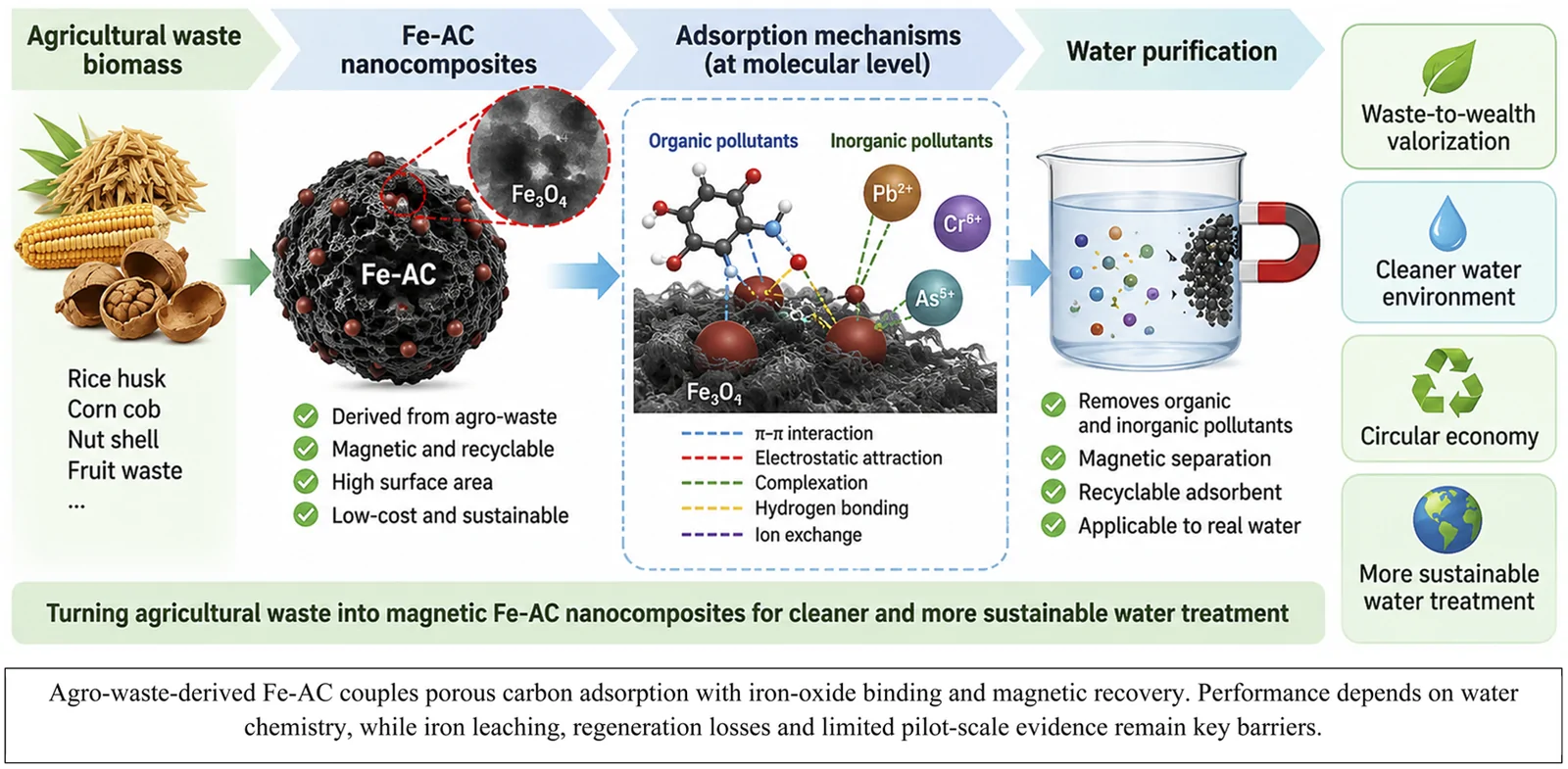

## 1 Introduction

### 1.1 Background on water contamination and the need for effective treatment methods

Water pollution is a significant environmental issue that not only impacts human health, but also the health of ecosystems and the provision of safe water. Many persistent pollutants, such as heavy metals, synthetic dyes, pharmaceuticals, pesticides, and other persistent pollutants are of particular concern because they can be found in relatively low concentrations and can be toxic, or are degraded slowly and are able to bioaccumulate in environmental compartments.^1^

Sources of organic contaminants such as pesticides, pharmaceuticals, personal-care products and industrial chemicals to aquatic systems include agricultural runoff, municipal wastewater, and industrial discharge. They are persistent, biologically active and may lead to endocrine disruption, developmental effects, ecological toxicity, and selection for antimicrobial resistance.^2^

Another important group of contaminants are synthetic dyes that are discharged from various textile and leather goods manufacturing, paper and paperboard manufacturing processes. Many dye molecules are chemically stable, not easily biodegradable and can be toxic, in addition to being visible. The presence of them can also diminish the light penetration and also disrupt the photosynthesis in receiving waters.^3^

Persistent elements include heavy metals and elements that are metalloids (not broken down by typical biological routes), including Pb, Cd, Hg, Cr and As. These species can enter surface and ground water from industrial activities, mining, smelting, manufacturing, and poor waste disposal practices, and can be significantly affected by speciation, pH, and competing ions in these environments, all of which will affect species mobility and treatment efficiency.^4^

The water-treatment technologies used for contaminant removal include sedimentation and filtration for particulate matter, as well as membrane separation, chemical treatment, biological treatment, ion exchange, and adsorption for dissolved contaminants. The suitability of these approaches depends on the nature of the contaminant and the physicochemical characteristics of the water. Among adsorptive treatment materials, iron oxide and its modified forms have been extensively investigated for the removal of contaminants such as arsenic from aqueous systems. In addition, plant-mediated biosynthesis has been explored as an alternative route for producing iron oxide nanoparticles with potential environmental remediation applications.^5,6^

Many biodegradable organic contaminants can be broken down by biological processes and/or low-energy treatment may be supported by these processes, but elemental metals cannot be broken down by biological processes. In the case of metals, separation, immobilization, precipitation, or a change in the oxidation state of the metal to a less mobile form is necessary. Thus, adsorption is very significant for waters that have persistent inorganic pollutants and poorly biodegradable organic pollutants.^7^

However, the simplicity of operation, flexibility, and capability to remove chemically diverse contaminants makes adsorption an attractive treatment technology among those available. Physisorption in an activated-carbon system is primarily the result of filling pores, van der Waals, hydrophobic and electrostatic forces; chemisorption may be owing to surface complexation, ligand exchange, ion exchange, coordination, precipitation and redox reactions. In Fe–AC composites, the carbon phase provides high surface area and aromatic domains, and the Fe-oxide phases provide Fe–OH and Fe–O sites that are suitable for the binding of metal ions and certain organic molecules. The relative importance of each mechanism will depend on the specific chemical properties of the contaminant, surface charge, pH, ionic strength, and the chemical state of the iron phase.^8,9^ The principal physical and chemical adsorption pathways, including surface interactions and binding at specific adsorption sites, are schematically illustrated in Fig. 1.

**Fig. 1:**
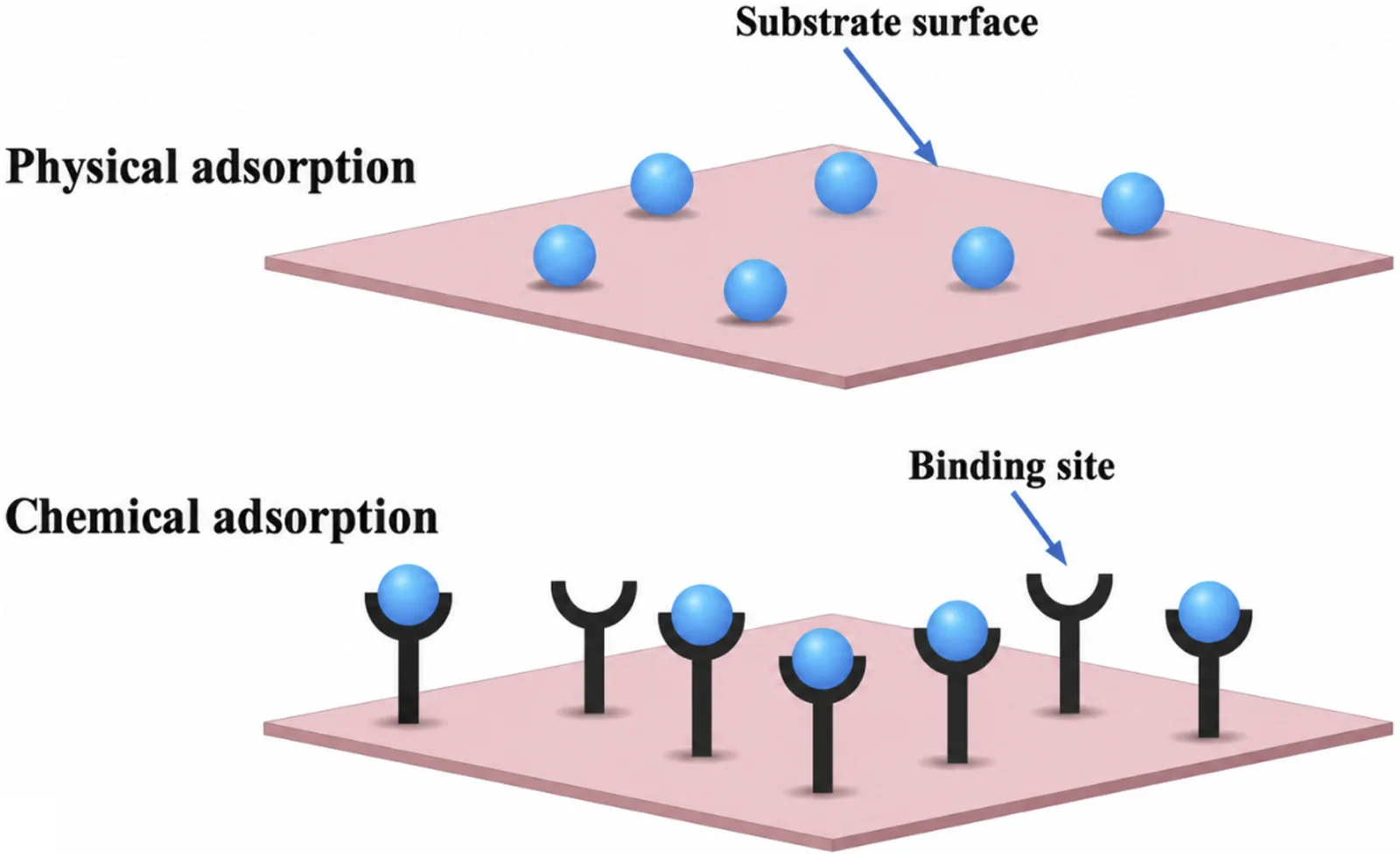
Schematic diagram of physical and chemical adsorption process.

Activated carbon is widely used in water treatment because its high specific surface area, tunable pore structure, and surface functional groups enable adsorption of chemically diverse contaminants. Its performance depends on precursor chemistry, activation conditions, pore-size distribution, and surface charge. Surface modification can add new binding functions, but any improvement should be evaluated together with changes in porosity, stability, regeneration, and production burden.^10,11^

Magnetite (Fe_3_O_4_) and maghemite (γ-Fe_2_O_3_) provide hydroxylated iron-oxide surfaces that can bind metal ions and oxyanions and, when sufficiently magnetic, facilitate adsorbent recovery. Loading these phases on activated carbon combines carbon porosity with Fe–OH/Fe–O binding sites. The improvement is not automatic: excessive iron loading can block pores, while poorly bound particles can leach during use or regeneration. Consequently, iron loading, phase identity, dispersion, and stability must be evaluated together with adsorption performance.^12,13^

Common routes for preparing iron oxide-grafted activated carbon (Fe–AC) include chemical precipitation, co-precipitation, sol–gel processing, and hydrothermal synthesis.^14^ Chemical precipitation and co-precipitation are comparatively simple and scalable, whereas sol–gel and hydrothermal routes can provide tighter control over particle size, crystallinity, and dispersion at the expense of greater process complexity and energy demand.^15^

To understand the physicochemical properties and performance of Fe–AC, characterization by XRD, SEM, TEM, BET surface-area analysis, FTIR, VSM, and XPS is commonly used.^16^ These methods provide complementary information on crystalline phase, morphology, nanoparticle dispersion, porosity, functional groups, oxidation state, and magnetic response, allowing synthesis conditions to be related to adsorption and recovery behavior.^17^Table 1 summarizes the principal characterization techniques and the material properties they are used to evaluate.

**Table 1:** Summary of principal characterization techniques applied to Fe–AC composites and the specific structural, surface, and magnetic properties each method elucidates

Technique	Measured parameter(s)	Purpose in Fe–AC studies	Typical output	Ref.
FTIR	Surface functional groups; Fe–O and O-containing groups	Confirm functionalization and changes after iron loading or adsorption	Bands assigned to O–H, C=O/C–O and Fe–O vibrations	18
XRD	Crystalline phase and crystallite structure	Confirm Fe_3_O_4_, γ-Fe_2_O_3_, Fe_2_O_3_ or other iron-containing phases	Phase-specific diffraction reflections	19
SEM/FESEM	Surface morphology and particle distribution	Assess pore-surface texture and visible iron-rich deposits	Micrographs; EDX can support elemental mapping	20
TEM	Nanoparticle size and dispersion	Resolve nanoscale iron-oxide domains and agglomeration	Particle-size and dispersion images	21
BET/N_2_ sorption	Specific surface area; pore volume; pore-size distribution	Quantify pore blocking or pore development after modification	BET area; micro/mesopore metrics	22
XPS	Surface elemental composition and oxidation state	Distinguish surface Fe chemical states and adsorption-induced changes	Fe 2p and relevant surface-element spectra	23
EDX/elemental analysis	Elemental composition and spatial distribution	Verify Fe loading and screen compositional uniformity	Fe content and elemental maps	24
Zeta potential	Surface charge as a function of pH	Interpret pH-dependent electrostatic attraction or repulsion	Zeta potential and point of zero charge	25
VSM	Saturation magnetization, coercivity, remanence	Assess magnetic recoverability and changes after functionalization	Magnetization curve and *M*_s_	26

Sorption of inorganic contaminants by Fe–AC can occur through two mechanisms: ion exchange and surface complexation, which are different processes. The difference between ion exchange and surface complexation is that the former is the replacement of counter-ions that are attached to charged surface groups while the latter consists of the formation of inner- or outer-sphere coordination complexes between surface functional groups and dissolved species. Ligand exchange, coordination, precipitation and redox-assisted immobilization can occur at the Fe–OH sites depending on the pH of the solution. The coordination, electrostatic interaction, pore filling, and pi–pi interaction are also contributed by carbonyl, carboxyl, hydroxyl and aromatic domains on the activated-carbon phase.^27^

Laboratory experiments showed the removal of metals, metalloids, dyes, pharmaceuticals, *etc.*, organic contaminants under controlled conditions in Fe–AC composites. The strength of evidence is significantly higher for batch tests using synthetic solutions as compared to continuous-flow treatment, real wastewater, and long duration field operation, however. Such reported performance must thus be read alongside that of pH, initial concentration, dose of adsorbent, contact time, water chemistry, regeneration history and analytical method, and cannot be interpreted based on adsorption capacity alone.^28^

There are significant challenges to address such as reproducible dispersion of iron-oxide, control of nanoparticle agglomeration, adsorption activity recovery during regeneration, minimizing iron leaching, and safe disposal of the contaminant-loaded spent sorbent. It is important to consider these issues in natural and industrial waters, where natural organic matter and other co-existing ions may compete for active sites, modify the surface charge, or form competitive complexes.^29^ While green synthesis and simplified characterization may help to decrease production burdens, the advantages of these methods must be established with standardized performance, leaching and life cycle metrics.^30^

Agricultural residues such as rice straw have also been investigated as modified and unmodified biosorbent precursors for water and wastewater treatment, highlighting their potential for the development of sustainable adsorption materials.^31^ The coupling of porous adsorption with chemically active surface sites and in many formulations, magnetic recoverability of biomass-derived activated carbon with iron-oxide phases is therefore promising. Recent reviews of sustainable carbon materials, porous adsorbents based on biochar, and carbon nanomaterials all highlight the need for real-world testing to consider mechanism, material stability, complex-water behavior, regeneration, and environmental impact in addition to the maximum adsorption capacity.^32,33^

### 1.2 Biosorbent activated carbon and its application in water treatment

Renewable or waste biomass like rice husk, coconut shell, sawdust, bagasse and other lignocellulosic wastes can be used to produce biosorbent activated carbon. Carbonization and physical or chemical activation creates a porous carbon matrix with both oxygen-bearing surface groups which are able to bind inorganic and organic contaminants. Agricultural residues have the potential to be used in waste valorization, however, the requirements for the handling of the feedstock, activation chemistry, energy requirements of the process, washing requirements, the need for regeneration, end-of-life management and the environmental benefits depend on the type of the process.^34,35^

Agricultural residues can be used as a variety of precursor materials for activated carbon and can help to limit biomass that goes to uncontrolled burning or disposal sites. They are however feedstock specific. Carbon yield, pore development, cost, and environmental performance are affected by ash content, lignocellulosic composition, moisture, transport distance and competing uses. These variables should be reported in comparing waste derived activated carbon from different studies.^36^

The typical production of activated carbon is carbonization and activation of the carbon. In the carbonization process, the prepared biomass is converted to a carbon rich char in an oxygen deficient environment and significant volatile matter is removed. While physical activation is usually performed with the aid of oxidizing gases like steam or CO_2_, chemical activation is usually treated with the aid of agents, *e.g.*, H_3_PO_4_ or KOH. The yield, pore size distribution, surface area, surface chemistry, washing demand and residual-chemical burden depend on the activation conditions.^37^

Iron-oxide loading can further functionalize biomass-derived carbonaceous materials; for example, Fe_3_O_4_-loaded amino-modified passion-fruit-shell nanocomposites have been applied for the adsorption of anionic Alizarin Red S dye from aqueous media.^38^ This carbon will have high surface area and interconnected micro and mesopores which will enhance accessibility to adsorption sites. However, adsorption performance should not be related to surface area alone as the availability of pores, surface charge, functional groups, contaminant size and solution chemistry influence adsorption capacity. Excessive loading can lead to a decrease in available carbon porosity, while iron-oxide modification can also increase the number of additional binding sites and magnetic recovery.

Heavy metals and metalloids (Pb, Cd, Hg, Cr and As) are persistent in aquatic systems and can bio-accumulate. These species can be removed from solution by activated carbon created from biomass by ion exchange, electrostatic attraction, coordination, and surface complexation. Iron-oxide modification can enhance the uptake of certain species by creating Fe–OH/Fe–O sites, but the predominant mechanism is pH-, species-, ligand-, and surface-chemistry dependent uptake of the species.^39,40^

Many textile, paper and leather effluents contain synthetic dyes which are persistent contaminants. The uptake of activated carbon can be achieved by pore filling, hydrophobic interactions, electrostatic attraction, hydrogen bonding, and π–π interactions. Selected molecules may be able to coordinate or experience charge-dependent interactions with iron-oxide sites. A typical Fe modified carbon system and the simultaneous existence of carbon and iron related adsorption pathways are shown in Fig. 2.^41,42^

**Fig. 2:**
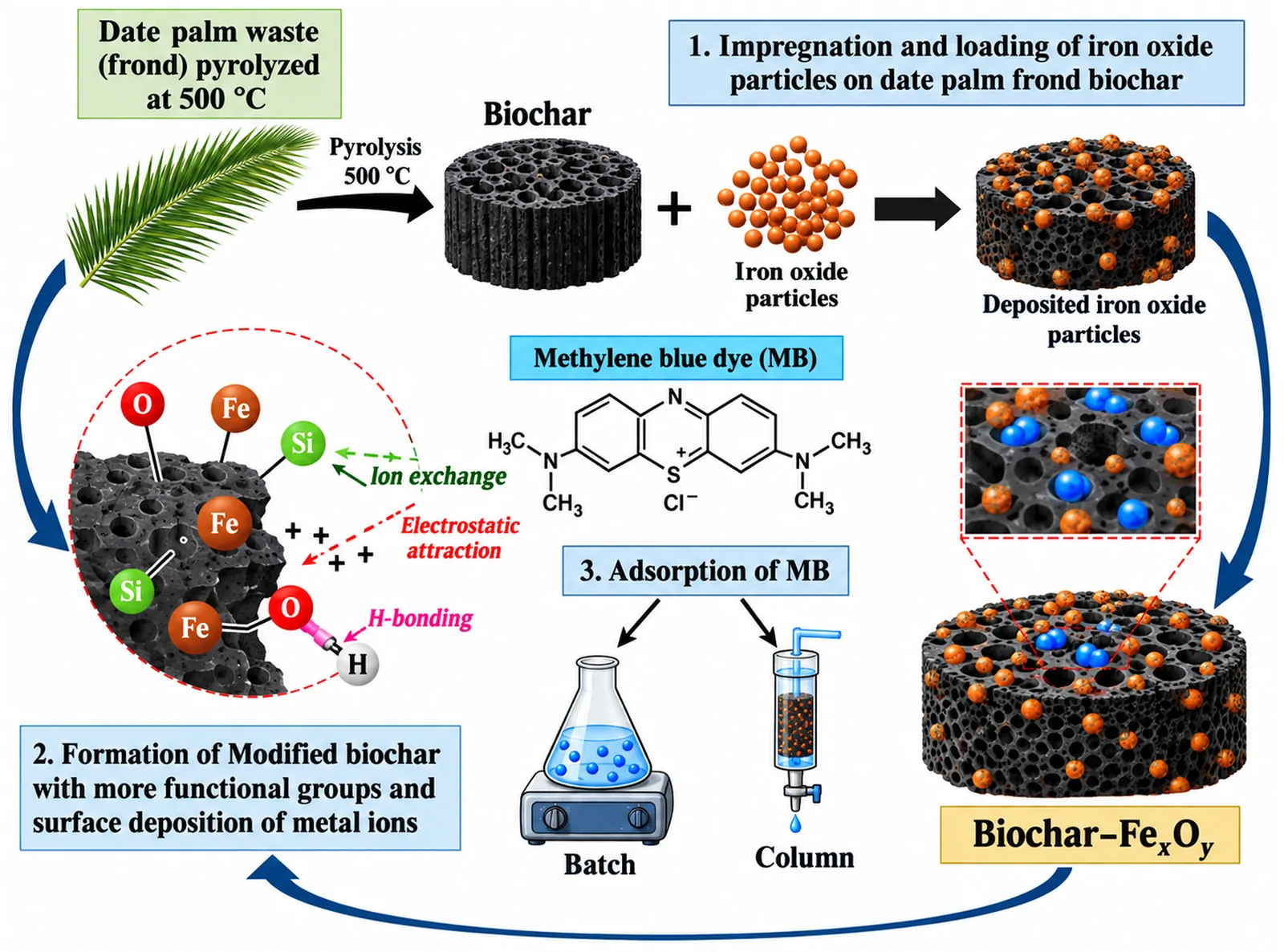
Representative Fe-modified carbon system illustrating preparation and adsorption pathways for an organic dye. The figure is used only to illustrate the coexistence of carbon–surface interactions and iron-oxide surface sites; photocatalytic pathways are considered only when experimentally demonstrated. Reproduced from ref. 43 with permission from Elsevier, Sudarmono *et al.*, “Magnetically separable and reusable Fe_3_O_4_/chitosan nanocomposites green synthesized utilizing Moringa oleifera extract for rapid photocatalytic degradation of methylene blue,” *Results Chem.*, 2024, **7**, 101245, doi: https://doi.org/10.1016/j.rechem.2023.101245, copyright 2024. License number: 6067060463082.

The adsorption of dyes and other organic molecules on Fe–AC are dependent on various molecule properties such as molecular charge, aromaticity, hydrophobicity, pore accessibility, surface functional groups, and solution pH. The electrostatic attraction is favoured when both the surface of the adsorbent and the adsorbate are oppositely charged, this contribution is dependent on the point of zero charge of the composite and the acid–base properties of the adsorbate. The aromatic dye molecules can also be able to bind to the graphitic domains by pi–pi stacking and hydroxyl, amino and carboxyl groups can hydrogen bond or coordinate with the domains. As a result, no overall trend can be established for the variation in pH for cationic, anionic and zwitterionic pollutants.^44^ The principal mechanisms governing heavy-metal and metalloid uptake by Fe–AC, including electrostatic attraction, ion exchange, surface complexation, precipitation, and redox transformation, are summarized schematically in Fig. 3.

**Fig. 3:**
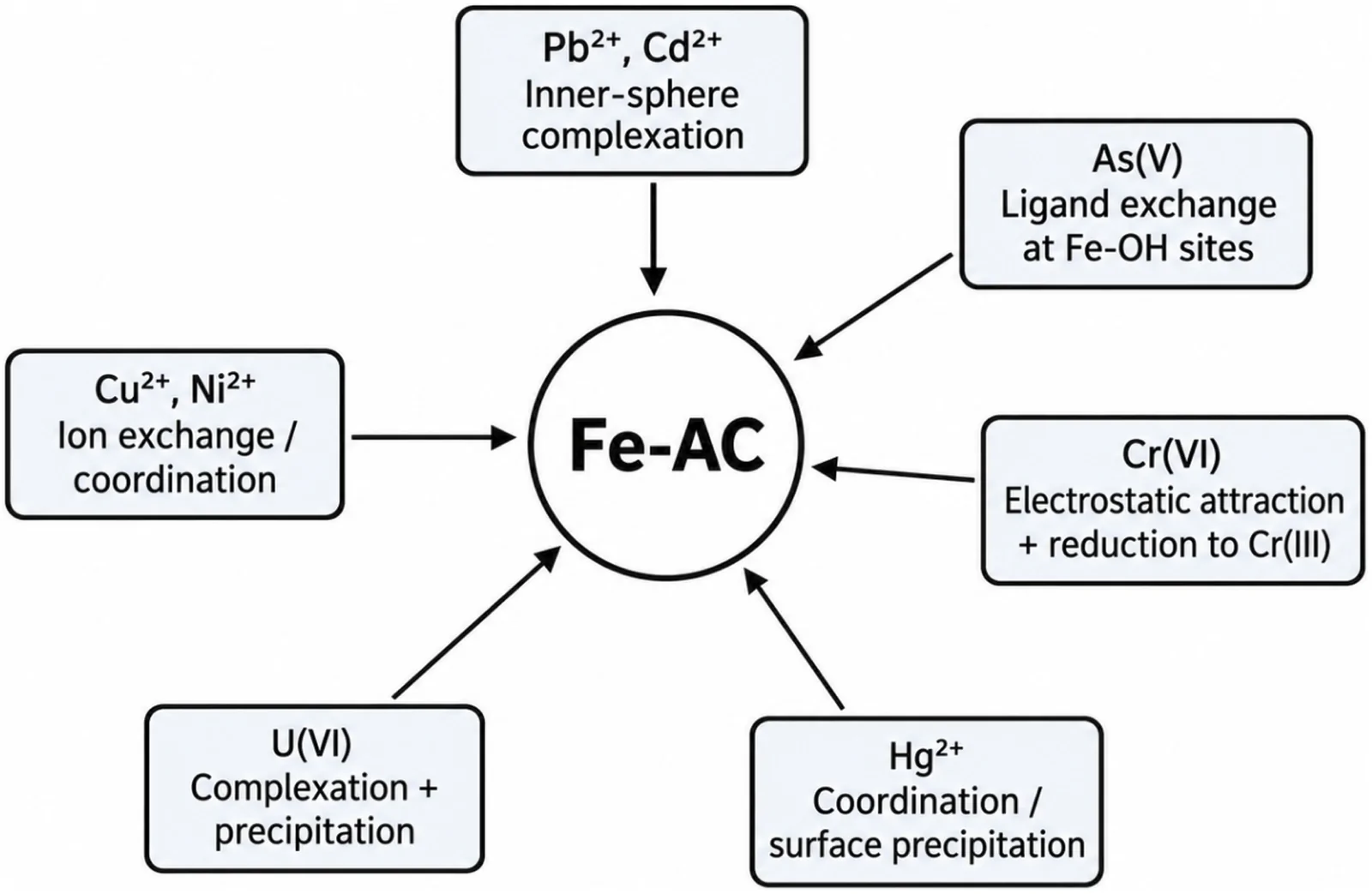
Principal molecular and interfacial mechanisms involved in heavy-metal and metalloid uptake by Fe–AC, including electrostatic attraction, ion exchange, inner-sphere complexation, surface precipitation, and redox transformation.

Organic chemicals (including pesticides, pharmaceuticals, phenols, and industrial chemicals) can also be found in water and are often not efficiently treated by traditional biological systems. A great many of these compounds can be bound to activated carbon by pore filling, hydrophobic interactions, hydrogen bonding, and π–π interactions. For the case of functional groups that are compatible with the Fe–O coordination, additional Fe–O coordination or redox pathways can contribute; however, the relative contribution of these pathways needs to be established for each contaminant and not assumed.^45^

Sustainability of biomass-derived activated carbon is not guaranteed by its intrinsic properties, but rather depends on the system under consideration. However, some of the benefits of using waste-derived feedstocks are offset by the need for carbonization, activation, washing, drying, production of iron salts, regeneration, transportation and management of the spent feedstock. Environmental claims should be tied to defined process boundaries and treatment performance, not source of feedstock.^46^

Regeneration can increase the life of the adsorbent service and can decrease the need for fresh adsorbent. The different regeneration methods (thermal, chemical, microbiological, microwave-assisted *etc.*) involve different desorption or destruction mechanisms, and require different levels of energy and reagent and different secondary-waste loads. When evaluating regeneration performance, all the following items may be reported together: capacity retention, carbon mass loss, pore-structure change, iron loss, and regenerant disposal.^47–49^

Reproducibility is a big practical problem. The composition of biomass, ash content, carbonization conditions, activating-agent ratio, washing, and iron-loading procedures can differ significantly from one study to another, and can affect pore structure, surface chemistry and adsorption capacity. Characterization and batch quality criteria should then be standardized before any laboratory performance could be scaled up to larger production volumes.^50,51^

Spent Fe–AC may not be considered as non-hazardous waste and may contain high levels of hazardous contaminants. Regeneration may lead to postponement of disposal, but end-of-life (EoL) management still needs to consider the need for a leaching assessment and selection of an appropriate recovery, stabilization, controlled thermal treatment, or disposal path. Reuse in other product is suitable provided contaminant release and long-term environmental safety are proven.^50,51^

Agro-waste derived activated carbon is one of the potentially useful precursors that can be used in water-treatment adsorbents due to their porous structure and use of renewable or waste-derived feedstocks. It is not possible to conclude its practical sustainability by the nature of the feedstock used. Reproducibility of the precursor, activation yield, reagent requirement, energy use and regeneration, contaminant leaching, and end-of-life disposal need to be carefully examined in an integrated assessment.^50,51^

### 1.3 Grafting of iron oxide nanoparticles onto biosorbent surfaces

Grafting iron oxide onto biomass-derived activated carbon can increase affinity for selected contaminants by introducing Fe–OH/Fe–O binding sites and, in magnetic formulations, by enabling external-field recovery. The extent of improvement depends on iron phase, loading, dispersion, carbon pore preservation, and water chemistry. Reported gains should therefore be interpreted against the corresponding unmodified carbon and with explicit measurement of iron stability and pore-volume change.^52^

### 1.4 Scope and objectives

This review evaluates the synthesis, characterization, adsorption behavior, regeneration, and application constraints of iron oxide-grafted agro-waste-derived activated carbon for water treatment. The scope is restricted to activated-carbon or closely related biomass-carbon supports containing iron-oxide or iron-oxyhydroxide phases that contribute directly to contaminant uptake, redox activity, or magnetic recovery. Non-iron metal oxides and unsupported adsorbents are discussed only when needed for contextual comparison.

The objectives are to compare major synthesis routes in terms of phase control, particle dispersion, energy and reagent requirements, scalability, and potential environmental burden; to relate characterization results to material structure and adsorption behavior; and to distinguish the mechanisms governing inorganic and organic contaminant removal.

Particular emphasis is placed on molecular-level interactions at Fe–OH, Fe–O, oxygenated carbon, and aromatic surface sites; on the effects of pH, contaminant speciation, competing ions, and natural organic matter; and on the extent to which batch adsorption results can be translated to regeneration, continuous-flow, and real-wastewater conditions.

Environmental and economic implications are assessed using published qualitative and quantitative evidence. Because functional units, system boundaries, energy mixes, precursor assumptions, and end-of-life scenarios differ substantially among available studies, the sustainability discussion is treated as a critical evidence synthesis rather than as a stand-alone quantitative life-cycle assessment.

### 1.5 Review methodology and literature search strategy

The Preferred Reporting Items for Systematic Reviews and Meta-Analyzes (PRISMA) framework was used to organize literature identification and screening, with adaptations for a materials-science review. The core systematic search covered Web of Science Core Collection, Scopus, PubMed, and Google Scholar for publications from January 2010 to December 2024. A targeted citation update was subsequently used to incorporate highly relevant 2025–2026 reviews and recent primary studies for contextual and mechanistic discussion. These post-search publications were not used to alter the screening counts reported for the core 2010–2024 corpus.

The search term used was: (iron oxide OR Fe_2_O_3_ OR Fe_3_O_4_ OR magnetite OR maghemite) AND (activated carbon OR biosorbent OR biochar) AND (water treatment OR adsorption OR heavy metal OR dye removal OR pharmaceutical). The following variations of the main terms were used for secondary search: Fe–AC composite, magnetic adsorbent water purification, iron oxide grafted activated carbon, Fe_3_O_4_@AC. Boolean operators (AND and OR) and truncation symbols were used to increase recall.

The inclusion criteria were: (i) peer-reviewed original research or review articles relevant to iron oxide-grafted/loaded activated carbon or closely related biomass-derived carbon composites; (ii) evidence on synthesis, characterization, adsorption, regeneration, leaching, or water-treatment performance; and (iii) sufficient experimental or mechanistic detail to support the point for which the study was cited. Studies based exclusively on non-iron metal oxides, unsupported iron salts, or materials without a carbon support were excluded from Fe–AC performance comparisons. Zero-valent iron systems were not treated as Fe–AC unless an iron-oxide phase was explicitly demonstrated and functionally relevant.

After duplicates were deleted, the initial database search resulted in 1847 records being returned. After screening titles and abstracts for relevance against the inclusion criteria, 312 full-text articles were assessed. Of these 183 met all inclusion criteria and are the basis for this review. An additional 24 references were found by hand searching from key review papers and were added where they contained unique mechanistic or performance data not retrieved from the database search.

#### 1.5.1 Scope and contribution

Existing reviews address magnetic iron-oxide adsorbents, biomass-derived carbon materials, heavy-metal uptake, and broader environmental applications of porous materials. The two additional 2026 reviews recommended during peer review were also examined: one evaluates uranium separation by biochar-based porous materials and the other reviews framework materials for nuclear-waste treatment.^34,35^ These papers are used only for directly relevant concepts such as pore/functional-group design, metal or radionuclide binding, mechanistic validation, and translation to complex waters; they are not treated as Fe–AC performance studies. The present review integrates these dimensions specifically for agro-waste-derived Fe–AC systems by linking synthesis and structure to contaminant-specific mechanisms, regeneration, matrix effects, scale-up barriers, iron-leaching risk, and the strength of available sustainability evidence.^32,35^ A comparative overview of the present review and closely related published reviews, highlighting differences in Fe phase, precursor type, pollutant coverage, life-cycle assessment, scale-up analysis, and mechanistic depth, is presented in Table 2.

**Table 2:** Comparing the present review against closely related published reviews. HM = heavy metals; pharma = pharmaceuticals; LCA = life-cycle assessment; AC = activated carbon

Review scope/focus	Fe phase	AC precursor type	Pollutant classes covered	LCA included	Scale-up analysis	Mechanistic depth
Magnetic iron oxide NPs for heavy-metal removal	Fe_2_O_3_, Fe_3_O_4_	Not specified	HM only	No	Partial	Moderate
Activated carbon composites for organic pollutants	Not specified	Commercial AC	Dyes, pharmaceuticals	No	No	Low
Biochar-iron oxide composites for water treatment	Fe_3_O_4_	Biochar only	HM, dyes	Partial	No	Moderate
Biosorbent adsorbents from agricultural waste	Not applicable	Agro-waste AC	HM	No	No	Low
Fe–AC composites: synthesis and adsorption (2020–2024)	Fe_2_O_3_/Fe_3_O_4_	Mixed	HM, dyes	No	No	Moderate
Present review	Fe_2_O_3_, Fe_3_O_4_, γ-Fe_2_O_3_	Biomass/agro-waste AC and closely related biomass carbon	HM, dyes, pharma, pesticides, emerging	Critical life-cycle evidence synthesis	Yes; engineering barriers and evidence gaps	High; contaminant-specific

## 2 Various synthesis routes for iron oxide grafted onto activated carbon

### 2.1 Raw materials used

Preparation of iron-oxide-grafted biosorbent activated carbon begins with selection and pretreatment of a suitable carbon precursor. Agricultural residues such as rice husk, coconut shell, sawdust, and other lignocellulosic wastes are carbonized and activated before or during iron loading. Iron salts such as FeCl_3_, FeSO_4_, or mixed Fe^2+^/Fe^3+^ precursors are then converted to iron-containing phases by impregnation, precipitation, thermal treatment, or related routes. Fig. 4 presents three representative alternative iron-loading procedures for powdered or granular activated carbon rather than a single sequential five-stage synthesis route.^53,54^

**Fig. 4:**
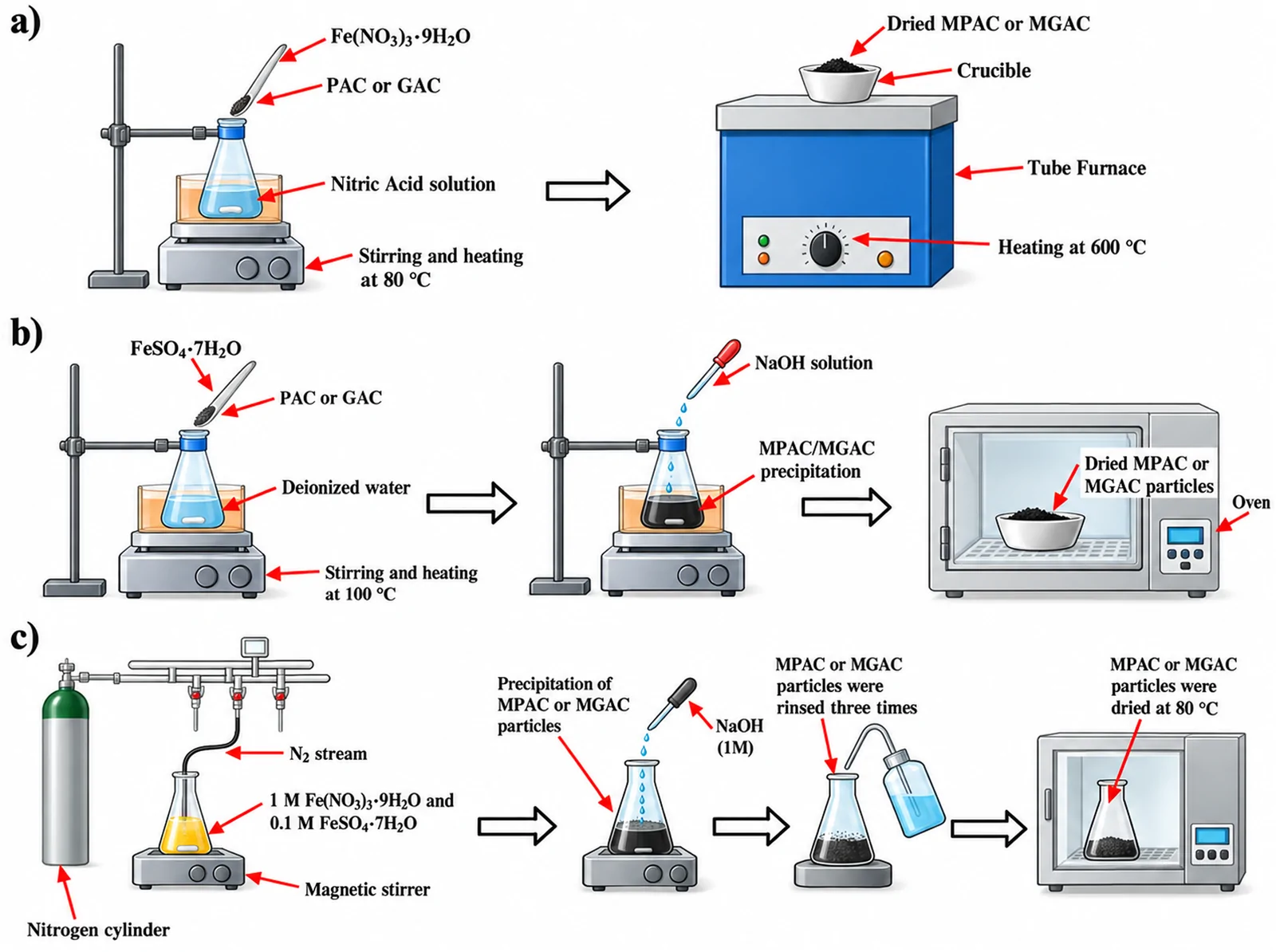
Representative alternative preparation routes for iron-modified powdered or granular activated carbon shown in the reproduced source: (a) Fe(NO_3_)_3_ impregnation followed by drying and thermal treatment; (b) FeSO_4_ treatment followed by NaOH-induced precipitation and drying; and (c) mixed Fe(iii)/Fe(ii) co-precipitation under N_2_ followed by repeated washing and drying. The three panels represent alternative preparation procedures, not sequential synthesis stages. Reproduced from ref. 54 with permission from Elsevier, B. E. Keshta *et al.*, “State of the art on the magnetic iron oxide Nanoparticles: Synthesis, Functionalization, and applications in wastewater treatment,” *Results Chem.*, 2024, **7**, 101388, doi: https://doi.org/10.1016/j.rechem.2024.101388, copyright 2024. License number: 6067090757827.

### 2.2 Overview of different synthesis techniques

Three main methods of synthesis of Fe–AC and related iron-oxide/carbon composites are reported: chemical precipitation, co-precipitation, sol–gel processing and hydrothermal or solvothermal synthesis. There are a number of differences in these routes: nucleation control, particle dispersion, crystallinity, Fe loading, energy demand, reagent use, wastewater generation, and scalability. Other process intensifying methods such as microwave and electrochemical method have also been studied.^55,56^ The generation of the supported iron phase to produce reactive species is not classified here as an application pathway, but is classified as a synthesis route.

#### 2.2.1 Chemical precipitation

Iron salts are reacted with a base or other precipitating agent in the presence of activated carbon to nucleate iron-hydroxide/iron-oxide phases on or in the activated carbon surface. The method is easily scalable and can be performed under relatively mild conditions, but uncontrolled nucleation may result in the generation of free particles, non-uniform loading, and agglomeration. Needs for washing materials and salts left over should be considered in the process evaluation.^57^

#### 2.2.2 Sol–gel processing

The hydrolysis and condensation of an iron precursor form a gel network, which is then dried and, if needed, subjected to a heat treatment. Compared to the ability to control composition and dispersion that can be achieved with sol–gel routes, performance is highly dependent on the chemistry of the precursors, the solvent system, the conditions under which the gel is formed, and the calcination temperature. Further solvent and thermal demands may also cause further process burden.^58^

#### 2.2.3 Synthesis under hydrothermal and/or solvothermal conditions

The precursors containing iron and the carbon is treated in a closed reactor under high temperature and autogenous pressure. The route can increase crystallinity and particle attachment but is subject to several constraints, including: pressure rated equipment, heating demand, batch operation and control of scale-up. Any claimed improvement in adsorption should be distinguished from the effects of increased crystallinity and pore blockage and altered surface chemistry.^59^

#### 2.2.4 Co-precipitation

The combination of activated carbon and certain controlled conditions (pH, temp and mixing) can be used to precipitate mixed Fe^2+^/Fe^3+^ salts. The technique is convenient and generally employed for the loading of magnetite since it requires relatively few unit operations and it is relatively fast. Important quality control variables are reproducible Fe^2+^/Fe^3+^ stoichiometry, oxygen exposure, particle nucleation, washing and iron attachment.^60^

#### 2.2.5 Additional approaches

The microwave assisted synthesis can reduce heating time and promote rapid nucleation, and electrochemical assisted deposition or other assisted deposition methods can control the spatial distribution of the iron formation. Instead of the length of the synthesis it is the total energy consumption, equipment intensity, yield, iron stability, and reproducibility that should be considered for their practical value.^61,62^

### 2.3 Comparative analysis of synthesis methods

#### 2.3.1 Efficiency

Hydrothermal and sol–gel routes can provide improved particle-size or crystallinity control, but higher structural control does not automatically produce higher treatment performance. The benefit depends on whether the resulting iron phase remains accessible without excessive blockage of the activated-carbon pore network.^63^

#### 2.3.2 Cost and scalability

Precipitation and co-precipitation generally require simpler equipment than pressure-based or multi-step routes and are therefore attractive for scale-up. Their apparent cost advantage remains dependent on iron-salt consumption, base demand, washing-water use, product yield, wastewater management, and quality control.

#### 2.3.3 Environmental implications

Hydrothermal and high-temperature routes can increase energy demand, whereas chemical routes can shift the burden toward reagent production, washing, and salt-containing effluent. Microwave-assisted routes may reduce processing time but require equipment-specific energy accounting. Table 3 therefore compares synthesis routes as engineering trade-offs rather than as a performance ranking.^64–70^

**Table 3:** Comparative analysis of synthesis methods for iron oxide-grafted biosorbent activated carbon

Synthesis method	Representative reagents/conditions	Advantages	Limitations	Scale-up interpretation	Ref.
Chemical precipitation	Fe salt + base; low-to-moderate temperature	Simple unit operations; flexible iron loading	Free-particle nucleation, agglomeration, washing demand	Potentially high if nucleation and washing are controlled	57
Sol–gel	Iron precursor + hydrolysis/condensation; drying/calcination as required	Good compositional and dispersion control	Solvent/reagent demand; gelation sensitivity; added thermal step	Low to moderate	58
Hydrothermal/solvothermal	Iron precursor + carbon in sealed reactor at elevated T/P	Good crystallinity and attachment control	Pressure equipment; batch heating; scale-up complexity	Moderate	59
Co-precipitation	Fe^2+^/Fe^3+^ salts + base under controlled pH/mixing	Rapid Fe_3_O_4_ formation; widely used; simple equipment	Oxidation sensitivity; pH/mixing control; salt-containing wash water	High when Fe loading and particle recovery are reproducible	65
Microwave-assisted	Iron precursor/carbon under microwave heating	Rapid volumetric heating; shorter processing time	Equipment-specific hot spots and scale-up energy accounting	Moderate; requires scale-up validation	61
Electrochemical/assisted deposition	Electrochemically generated/deposited iron phase on carbon support	Potential spatial control and lower bulk reagent use	Electrode/reactor complexity; electricity demand	Low to moderate; evidence remains limited	69

The synthesis routes in Table 3 should not be ranked solely by adsorption capacity because precursor chemistry, iron loading, activation conditions, particle size, pH, and test conditions differ among studies. Co-precipitation is attractive for scale-up because it combines comparatively mild conditions with rapid iron-oxide formation, whereas hydrothermal and sol–gel methods can provide tighter structural control at the cost of higher energy, equipment, or reagent requirements. Assisted routes remain promising but require direct assessment of yield, leaching, reproducibility, and process energy.^63–70^

## 3 Applications of iron oxide-grafted activated carbon (Fe–AC) in water treatment

### 3.1 Overview of contaminants removed

Fe–AC composites have been evaluated for heavy metals and metalloids, dyes, pharmaceuticals, pesticides, phenols, and other emerging contaminants. The controlling interactions differ substantially among these classes. Inorganic ions are governed primarily by aqueous speciation and surface coordination, whereas organic molecules are additionally influenced by molecular size, aromaticity, hydrophobicity, pKa, and pore accessibility. Performance data are therefore considered by contaminant class and interpreted together with experimental conditions.^71^

### 3.2 Removal of heavy metals and metalloids

Heavy metals and metalloids such as Pb^2+^, Cd^2+^, Cr(vi), Hg^2+^, As(v), and U(vi) can be retained by Fe–AC through combinations of inner-sphere complexation, ligand exchange, ion exchange, electrostatic attraction, precipitation, and redox transformation. Fe–OH sites can coordinate cations or exchange ligands with oxyanions, while redox-active Fe^2+^ or carbon functionalities can contribute to Cr(vi) reduction under suitable conditions. The reviewer-recommended studies on magnetic activated carbon and functionalized activated carbon have been incorporated where they directly support heavy-metal uptake, reusability, and real-wastewater performance.^72–84^ Representative Fe–AC systems investigated for the removal of heavy metals and metalloids, together with their reported adsorption performance, experimental context, and proposed mechanisms, are summarized in Table 4.

**Table 4:** Representative Fe–AC performance for heavy metals and metalloids under reported experimental conditions

Adsorbent/study	Target ion(s)	Reported performance	Key experimental context	Mechanistic interpretation	Ref.
Fe_3_O_4_ NPs@AC@C_4_H_8_SO_3_H from pistachio-shell AC	Pb^2+^, As(iii), Cd^2+^	*q*_max_ reported for Pb^2+^ 147.05, As(iii) 151.51 and Cd^2+^ 119.04 mg g^−1^	Magnetic functionalized AC; batch adsorption with pH and dose optimization	Specific functional groups plus magnetic-AC surface binding	72
Tartaric-acid-functionalized magnetic activated carbon	Cr, Co, Pb	*q*_max_ values reported in the source for all three metals; reusable over repeated cycles	Pistachio-shell-derived magnetic AC; multivariable adsorption study	Chelation/complexation on functionalized magnetic carbon	75
Sulfamic-acid-functionalized magnetic activated carbon	Cu, Fe, Zn, Ni in industrial copper-mine wastewater	Best multimetal removal reported near pH 6.5; reuse evaluated for up to seven cycles	Real industrial wastewater rather than a single-solute matrix	Functional-group complexation plus magnetic recovery	76
Iron oxide-maize-shell activated biocarbon nanocomposite	Cd^2+^	High Cd^2+^ uptake reported for the Fe-modified biomass carbon	Biomass-derived carbon; equilibrium/kinetic evaluation	Surface complexation and ion-exchange contributions	77
Activated carbon/alginate/Fe_3_O_4_ magnetic nanocomposite	Pb^2+^	High Pb^2+^ removal reported in aqueous media	Composite magnetic sorbent; pH-dependent adsorption	Coordination/electrostatic binding on functionalized composite	78
Fe_3_O_4_-loaded activated carbon from castor seed shell	Cd^2+^	Equilibrium and kinetic adsorption demonstrated	Biomass-derived activated carbon with Fe_3_O_4_ loading	Fe-associated binding plus carbon adsorption sites	52
Tannic-acid/Fe_3_O_4_-modified corn-straw biochar	Cr(vi)	94.02% removal at pH 3, *C*_0_ 10 mg L^−1^, dose 1.0 g L^−1^; 81.44% after four cycles	Acidic-water study with explicit Fe-leaching measurements	Adsorption coupled with Fe-assisted reduction/complexation	81
AC@Fe_3_O_4_–NH_2_–COOH from walnut-shell activated carbon	Hg^2+^	Hg^2+^ removal demonstrated under saline conditions	Functionalized magnetic AC; salinity included in the design	Coordination to surface donor groups on magnetic carbon	84

Table 5 collates experimental variables that materially affect apparent adsorption performance. The comparison shows why published *q*_max_ values can appear contradictory without representing a true conflict in material quality. A high fitted capacity obtained at high initial loading is not directly comparable with removal measured at low *C*_0_, different pH, a different adsorbent dose, or a real wastewater matrix. Studies that report regeneration, iron leaching, salinity tolerance, or real-water operation provide additional practical evidence that cannot be inferred from *q*_max_ alone.

**Table 5:** Cross-study comparison of operating conditions and practical evidence for representative Fe–AC and closely related magnetic carbon adsorbents. NR = not reported in the accessible source summary; *q*_max_ values are study-specific and should not be ranked without harmonized conditions

Material/study	Pollutant	pH	Initial concentration/matrix	Adsorbent dose	Contact/mode	Reported outcome	Matrix/leaching/scale evidence	Ref.
Fe_3_O_4_ NPs@AC@C_4_H_8_SO_3_H	Pb^2+^/As(iii)/Cd^2+^	Optimized in source	Aqueous single-solute tests	Optimized in source	Batch	*q*_max_ 147.05/151.51/119.04 mg g^−1^	Magnetic separation; illustrates high fitted capacities under optimized batch conditions	72
TAR@magnetic AC	Cr/Pb/Co	Optimized in source	Aqueous heavy-metal tests	Optimized in source	Batch	*q*_max_ about 86.55/83.30/83.72 mg g^−1^ reported	Five-cycle reuse reported; lower fitted values than some studies do not imply inferior practicality	75
Functionalized magnetic AC from pistachio shell	Cu/Fe/Zn/Ni	∼6.5 optimum reported	Industrial copper-mine wastewater	NR	Batch/real water	Multimetal removal demonstrated	Real industrial copper-mine wastewater; competitive multimetal adsorption and reuse up to seven cycles were evaluated	76
Tannic-acid/Fe_3_O_4_ corn-straw biochar	Cr(vi)	3.0	10 mg L^−1^	1.0 g L^−1^	Batch; adsorption–desorption cycling	94.02% removal; 81.44% after four cycles	Coexisting Mg^2+^, Ca^2+^, Na^+^, CO_3_^2−^ and SO_4_^2−^ reduced Cr(vi) removal by 6.75–10.50%. Fe release was 0 mg L^−1^ at pH 3, 0.106 mg L^−1^ at pH 2.5, and >2.1 mg L^−1^ at pH 1–2	81
AC@Fe_3_O_4_–NH_2_–COOH	Hg^2+^	7	Saline solution; salinity 25 000 ppm	NR	Batch	Langmuir *q*_max_ ≈80 mg g^−1^	Demonstrates adsorption under high salinity rather than deionized-water conditions	84
Activated carbon/Fe_3_O_4_ nanocomposite	Cr(vi)	3.0	12 mg L^−1^ Cr(vi), aqueous solution	1.5 g L^−1^	Batch; 40 min	Optimized Cr(vi) removal under response-surface conditions	Directly demonstrates joint dependence on pH, initial concentration, adsorbent dose, and contact time	80

### 3.3 Removal of organic contaminants (dyes, pharmaceuticals, pesticides, and emerging pollutants)

Organic contaminants interact with Fe–AC through mechanisms that differ from those governing metal-ion uptake. Aromatic dyes, pesticides, and pharmaceuticals can be retained by pore filling, hydrophobic partitioning, π–π interactions, hydrogen bonding, and pH-dependent electrostatic attraction or repulsion. Iron-oxide phases may contribute Lewis-acid/base interactions, Fe–O coordination, or redox activity for compatible functional groups. Published Fe_3_O_4_/activated-carbon studies also show that iron fraction, pH, carbon pore structure, and regeneration conditions can materially change uptake, so mechanism assignments should be supported by characterization rather than inferred from kinetic-model fit alone.^85–98^

### 3.4 Case studies and experimental results

Case studies consistently show that iron modification can improve adsorption when new Fe-associated binding sites or magnetic-recovery functions are added without excessive loss of accessible carbon porosity. The size of the improvement is contaminant- and formulation-specific, and some systems rely predominantly on the carbon phase while iron oxide primarily facilitates recovery. Representative biomass-carbon and magnetic activated-carbon studies also demonstrate that precursor choice and preparation route strongly affect the resulting surface properties.^99–101^

For example, iron-modified pistachio-shell activated carbon achieved very high Acid Red 14 uptake under the tested concentration and pH ranges, whereas Fe_3_O_4_-based magnetic carbons used for methylene blue show different fitted capacities because their precursors, pore structures, temperatures, and test conditions differ.^18,88,97,100,101^ These differences should be interpreted as condition-dependent evidence rather than as a simple ranking of adsorbents. Table 7 therefore reports kinetic and thermodynamic observations together with the experimental context.

**Table 6:** Representative adsorption evidence for organic contaminants on Fe–AC or closely related magnetic activated-carbon systems. Values are reported under study-specific conditions and are not direct performance rankings

Adsorbent	Organic contaminant	Reported performance	Key operating conditions/matrix	Interpretation	Ref.
MnFe_2_O_4_-supported activated carbon (closely related magnetic carbon)	Acetochlor pesticide	Adsorption capacity ≈226 mg g^−1^ at 0.2 g L^−1^ adsorbent and 25 °C; equilibrium ≈10 h; regenerated adsorption capacity decreased by ≈50%	Aqueous acetochlor; MnFe_2_O_4_/AC mass ratio 1 : 2; pH, ionic strength, and humic-acid concentration were varied	Hydrogen bonding, π–π interactions, and pore filling; the reported adsorption was comparatively insensitive to the tested matrix variations	85
Magnetic activated carbon with controlled Fe_3_O_4_ fraction	Sulfamethoxazole	MPAC6 (14.3 wt% Fe_3_O_4_) showed Langmuir *q*_max_ = 173.0 mg g^−1^; 92.3% SMX removal remained in the fifth cycle	Five Fe_3_O_4_ : PAC ratios (1 : 1 to 1 : 8) were compared; strong SMX removal was reported at pH 2–4	Demonstrates the trade-off between magnetic loading and accessible carbon adsorption sites	86
Fe_3_O_4_-charcoal composite	Methylene blue	Batch *q*_max_ 97.49 mg g^−1^; fixed-bed capacity 90.85 mg g^−1^	Batch and fixed-bed systems; equilibrium ≈3 h in batch; adsorbent load 1.0 g L^−1^; microwave irradiation followed by methanol elution used for regeneration	Provides continuous-flow evidence together with regeneration and magnetic recovery	87
Fe_3_O_4_-waste-paper activated carbon	Methylene blue	*q*_max_ about 101 and 93 mg g^−1^ for two composites	Optimum pH 6; temperature dependence evaluated up to 315 K; contact time, dose, and initial MB concentration were varied	Langmuir fit and pseudo-second-order kinetics reported	88
Fe-oxide-modified activated carbon	Crystal violet	Condition-dependent dye adsorption	Batch aqueous study; detailed pH, initial concentration, dose, and contact-time values were not identifiable from the verified accessible source record	Carbon pore interactions plus Fe-associated surface effects	89
Chitosan-Fe_3_O_4_/activated carbon composite	Methylene blue and Congo red	98.1% MB and 100% CR removal under optimized conditions; Langmuir *q*_max_ = 11.02 mg g^−1^ for MB and 256.41 mg g^−1^ for CR	pH 6.50; contact time 60 min; adsorbent dosage 0.05 g; simultaneous MB/CR aqueous system	Illustrates contaminant-specific pH response in a multifunctional magnetic composite	92
Fe_3_O_4_/AC/zeolite nanocomposite	Tetracycline	85–87% removal at the design optimum; ≈90% in synthetic water and 82% in real pharmaceutical wastewater at the higher practical dose	pH 7; *C*_0_ = 100 mg L^−1^; 35 °C; 90 min; 1.0 g L^−1^ at design optimum and 2.0 g L^−1^ for synthetic/real-wastewater validation	Quantifies the performance decrease when the same material is transferred from synthetic solution to real pharmaceutical wastewater	93

**Table 7:** Kinetic, isotherm, and thermodynamic observations for representative in-scope iron-modified or Fe_3_O_4_/activated-carbon adsorbents

Adsorbent	Contaminant	Reported capacity (mg g^−1^)	Experimental conditions/isotherm	Kinetic model	Thermodynamic/practical observation	Reference
Iron-modified pistachio-shell AC	Acid Red 14	>516	AR14 20–150 mg L^−1^; pH 3–10; dose 0.1–0.3 g L^−1^; contact time 5–60 min. Freundlich: *K*_f_ = 152.87; *n* = 4.61	Pseudo-second-order	Spontaneous and exothermic; Δ*H*° = −41.86 kJ mol^−1^	18
Fe_3_O_4_-waste-paper AC	Methylene blue	101 and 93	Optimum pH 6; temperature dependence evaluated to 315 K; initial concentration, dose, and contact time varied. Langmuir model provided the best fit	Pseudo-second-order	Endothermic and spontaneous over the studied range	88
AC/Fe_3_O_4_	Methylene blue	312.8	pH 2–9; dose 0.05–0.5 g L^−1^; 298–318 K; contact time 5–180 min; *C*_0_ 50–500 mg L^−1^. Langmuir/Freundlich models evaluated	Pseudo-second-order; ANN used for process modelling	Thermodynamic and kinetic parameters reported	97
Fe_3_O_4_-activated charcoal from Ihau fruit peel	Methylene blue	8.7014	Optimum pH 9; contact time 45 min; Langmuir model. Initial concentration and adsorbent dose were not identifiable from the verified accessible source summary	Study-specific kinetic analysis	Optimum pH 9; 45 min; spontaneous/endothermic physical adsorption reported	100
Fe_3_O_4_-activated carbon from egg-rack-derived carbon	Methylene blue	126.043	pH 7; *C*_0_ = 60 mg L^−1^; 10 mg adsorbent in 25 mL (0.4 g L^−1^); contact time 90 min	Batch adsorption study	Fe_3_O_4_-AC showed greater MB uptake than the unmodified activated carbon; magnetic response supported separation	101

### 3.5 Comparison with conventional water treatment techniques

Iron oxide-grafted biosorbent activated carbon can complement conventional activated carbon, ion-exchange media, and membrane processes, but no single technology is universally superior. Conventional activated carbon is effective for many organic contaminants, while iron-oxide grafting can add affinity for selected metals, oxyanions, and polar compounds and can facilitate magnetic recovery. These gains must be balanced against pore blockage, iron-salt and washing demand, Fe leaching, particle handling, and regeneration losses.^102^

Ion exchange can provide high selectivity for ionic contaminants but requires regenerant chemicals and management of concentrated waste streams. Reverse osmosis and related membrane processes provide broad rejection but require pressure, fouling control, concentrate management, and membrane replacement. Fe–AC should therefore be compared using treatment-normalized metrics such as bed volumes treated, effluent quality, energy, chemical demand, adsorbent lifetime, regeneration burden, and cost per unit of compliant water rather than batch *q*_max_ alone.^102,103^

### 3.6 Real-world applications and scalability

Most published Fe–AC evidence remains laboratory-scale and is dominated by batch tests using synthetic single-contaminant solutions. Continuous-flow column data, long-duration breakthrough studies, pilot-scale operation, and validation with real municipal or industrial wastewater are comparatively scarce. Claims of field readiness should therefore remain conditional until performance is demonstrated under realistic hydraulic loading, competing-ion concentrations, natural organic matter, variable pH, and repeated regeneration.

Scale-up is further constrained by reproducibility of biomass feedstocks, control of iron loading across large carbon batches, particle attrition, pressure drop in packed beds, recovery of fine magnetic particles, and management of iron and contaminant leaching. For practical deployment, Fe–AC should be evaluated against treatment targets using breakthrough capacity, bed volumes treated, effluent iron concentration, regeneration efficiency, and total treatment cost rather than batch *q*_max_ alone.

### 3.7 Matrix effects, iron leaching, and continuous-flow constraints

Natural organic matter can occupy hydrophobic sites, coat iron-oxide surfaces, obstruct carbon pores, and complex dissolved target species. Phosphate, carbonate, sulfate, silicate, chloride, and competing cations can also alter surface charge or compete for Fe–OH sites. The available quantitative evidence demonstrates that these effects are material- and contaminant-specific. For tannic-acid/Fe_3_O_4_-modified corn-straw biochar, coexisting Mg^2+^, Ca^2+^, Na^+^, CO_3_^2−^, and SO_4_^2−^ reduced Cr(vi) removal by 6.75–10.50%; Fe release was 0 mg L^−1^ at pH 3, 0.106 mg L^−1^ at pH 2.5, and above 2.1 mg L^−1^ at pH 1–2.^81^ AC@Fe_3_O_4_–NH_2_–COOH retained an approximately 80 mg g^−1^ Langmuir capacity for Hg^2+^ at pH 7 in a saline solution containing 25 000 ppm salts.^84^ A closely related MnFe_2_O_4_/AC system showed little change in acetochlor adsorption across the tested pH, ionic-strength, and humic-acid ranges, although its adsorption capacity after regeneration decreased by approximately 50%.^85^Tables 5 and 6 retain these operating and matrix conditions alongside the reported performance.

Engineering validation remains much smaller than the batch synthetic-water literature. A Fe_3_O_4_-charcoal composite showed capacities of 97.49 mg g^−1^ in batch and 90.85 mg g^−1^ in a fixed-bed system and was regenerated by microwave irradiation followed by methanol elution.^87^ A Fe_3_O_4_/activated-carbon/zeolite composite removed approximately 90% tetracycline from synthetic water but 82% from real pharmaceutical wastewater at pH 7, 35 °C, 90 min, *C*_0_ = 100 mg L^−1^, and 2.0 g L^−1^ adsorbent.^93^ Functionalized magnetic activated carbon has also been evaluated in industrial copper-mine wastewater with multimetal competition and repeated reuse.^76^ These results support reporting influent matrix, pH, natural organic matter, major co-ions, flow or empty-bed contact time, breakthrough behavior, dissolved Fe, regeneration efficiency, and capacity retention whenever practical performance is claimed.

## 4 Environmental and economic implications

### 4.1 Environmental benefits of using biosorbent activated carbon

The use of waste biomass as a carbon precursor for Fe–AC, and adding the Fe phase to enhance treatment efficiency, recovery, or service life of the adsorbent, can offer environmental benefits. The benefits are only realized when the system is activated, iron grafted, washed, dried and materials replaced, which introduces additional energy, chemical, water and residual-management costs.^50,96^

Environmental comparison should thus be based on the treatment performance and not on the feedstock or the maximum adsorption capacity of a batch. Quantities of water that meet a specified effluent criterion, adsorbent usage, regeneration frequency, release of dissolved-iron, energy and chemical usage, and quantities and hazards of secondary residuals are useful decision metrics.^47–50,96^

Magnetic recovery may help minimize solid/liquid separation difficulty, but cannot remove spent sorbent, regenerant solutions, desorbed contaminants, fines or dissolved iron. The residues must be recovered, stabilized, treated or disposed of properly based on the amount of contamination and the behavior of leaching.^47–50^

Without a clear functional unit, system boundary, and process inventory, broad statements of the low-carbon or carbon-negative, or environmentally superior nature of biomass Fe–AC are not warranted. Feedstock substitution is just a part of environmental profile.^50,96^

Environmental performance should be associated with measurable material properties, like carbon yield, pore structure, Fe loading, magnetic response, mechanical strength, leachable Fe, working adsorption capacity in a reference matrix and capacity after regeneration for material development. These are used to assess the modification alternatives for synthesis with both treatment benefit and process burden.^50,63^

The main environmental and/or economic trade-offs are summarized in Table 8, although the relative benefit of the options depends on the feedwater composition, energy mix, regeneration strategy, material lifetime, and end-of-life assumptions.^50,96^

**Table 8:** Qualitative environmental and economic comparison of Fe–AC with selected treatment materials and processes

Parameter	Fe-grafted biomass AC	Commercial activated carbon	Ion-exchange media	RO/NF membranes	Evidence note/reference
Primary material basis	Biomass carbon + iron salt/reagents	Coal, wood, coconut shell or other commercial precursors	Synthetic resin matrix with functional groups	Polymeric/inorganic membrane materials	Feedstock alone does not determine life-cycle burden^50^
Dominant process burden	Carbonization/activation, Fe grafting, washing, drying	Carbonization/activation; periodic replacement/regeneration	Resin synthesis and chemical regeneration	Pressure energy, pretreatment, cleaning, concentrate handling	Compare using equivalent water-quality targets^102,103^
Regeneration	Possible; method and capacity retention are formulation-specific	Established thermal/chemical options for many GAC systems	Chemical regeneration common	Membrane cleaning/replacement rather than adsorbent regeneration	Regeneration benefit depends on energy, chemicals and residuals^47–49^
Secondary residuals	Spent sorbent, regenerant, possible Fe release and fines	Spent carbon and regeneration residuals	Concentrated regenerant waste	Concentrate and cleaning waste	Secondary waste is shifted, not eliminated
Matrix sensitivity	NOM, co-ions, pH and Fe stability can be critical	NOM and competitive organic adsorption important	Competitive ions affect selectivity	Fouling/scaling and feed composition are critical	Real-water validation is required
Potential advantage	Agro-waste valorization; added Fe sites; possible magnetic recovery	Mature supply chain and well-established operation	High ion selectivity	Broad rejection for many dissolved contaminants	Advantage is application-specific, not universal
Main uncertainty	Scale-up, long-term leaching, lifetime, TEA/LCA	Energy and regeneration assumptions vary by product	Regenerant consumption and resin end-of-life	Energy, fouling, concentrate disposal	Harmonized functional units are required^96^

### 4.2 Cost analysis of synthesis and application

Production cost is related to logistics of raw materials, carbon production yield, activation process, iron salt requirement, washing and drying, equipment, labor, quality control and production volume. The benefit of using agricultural residues is that the precursor can be purchased at a low cost, but the use of such residues does not necessarily lead to a low cost of the final adsorbent as process and post-treatment costs can become significant.^41,50^

Additional cost considerations for local sourcing include collection, drying, storage, contamination control, and competing uses of feedstocks, but can help to mitigate the burden on transport and waste handling. Agricultural residues should not be assumed to be free feedstock; the delivered and processed feedstock cost should be reported in the economic analysis.

Activation can be a significant cost and environmental factor. Physical activation involves continuous high-temp gas treatment, while chemical activation can lower some thermal requirement, but increases the cost of the activating agent, corrosion control, washing, recovery, and wastewater treatment. So the preferred route will be based upon yield, pore properties, reagent recovery, energy source and the desired treatment performance.^47–50^

The material and processing cost of iron-oxide grafting is iron salts, pH-control reagents, mixing, washing, drying and quality verification. While co-precipitation and precipitation are relatively straightforward, what is required for economic claims is the control of reagent use, yield, Fe retention, generation of wastewater, and batch reproducibility, not a qualitative description of the simplicity of the process.^52,63^

While economies of scale can lower unit costs due to better equipment utilization and bulk purchasing, they can also pose mixing, heat-transfer, washing, particle-recovery, and quality-control problems. Scale-up should be demonstrated experimentally, not from the perspective of laboratory simplicity.

The energy needed for carbonization, activation, drying and regeneration may be considerable. Process intensification, heat recovery, use of renewable electricity, improved carbon yield, and reduction in required activation temperature (when feasible) and use of reusable reagents are potential cost reduction measures, but must be evaluated using mass and energy balances.^47–50^

The cost of total synthesis depends on the scale of the synthesis and its location, and includes the costs of labor, infrastructure, environmental controls, analytical quality assurance, and occupational-safety requirements. Such costs should be accounted for in the techno-economic analysis along with capital depreciation and maintenance.

Drying, milling, particle sizing, granulation and packaging impact product performance and cost. Mechanical strength and particle size distribution are of special significance for fixed-bed operation, since too many fines will increase the pressure drop and loss of adsorbent.

Low-density biomass may have to be transported long distances, where transportation can become important. A proximity of biomass source to preprocessing, as well as transportation of densified intermediate or finished sorbent, can help to reduce logistics burden, but the proximity should be site-specific and should be tested in the sensitivity analysis.

Treatment options should be compared on an equal water quality basis for economic feasibility. Different capital, energy, chemical, residuals-management and replacement profiles exist for membranes, ion exchange, conventional activated carbon and chemical precipitation. It is therefore not enough to make comparisons based on adsorbent purchase price or *q*_max_.^102,103^

The extra binding sites, magnetic recovery, or regenerability of Fe–AC can lead to lower overall treatment burden in applications where the cost of Fe–AC is competitive. This advantage has not been universally agreed upon, and must be shown by cost per cubic meter of water that meets a specified effluent criterion.

The lifetime material demand may be reduced with regeneration, as long as the capacity is maintained throughout the cycles, but the cost of regenerant, regenerant heating, washing, contaminant recovery, Fe loss, and adsorbent mass loss should also be considered. The economic value of regeneration is only realized if the costs of regeneration are less than the avoided cost of replacement and disposal.^47–49^

The overall production cost is influenced by the various factors such as handling of precursor, activation, iron grafting, process yield, utilities, labour, quality assurance and scale. Agricultural wastes could be a good precursor base, but there is a need for sound mass, energy and cost data which can only be obtained on a site-specific basis to make such claims.^50^

Application cost encompasses the cost of the contactor or column equipment, pumping, adsorbent replacement, regeneration, analysis, residuals management and disposal. These data are not commonly reported together for Fe–AC and there is no evidence to definitively conclude that the cost of Fe–AC is less than established treatment technologies; however, the economic potential is supported.

## 5 Qualitative life-cycle and sustainability assessment

The environmental profile of Fe–AC is assessed qualitatively across feedstock acquisition, carbonization and activation, iron-oxide grafting, water-treatment use, regeneration, and end-of-life management. A quantitative life-cycle assessment requires a defined functional unit, system boundary, inventory, allocation method, impact categories, uncertainty analysis, and consistent comparison scenario. Because the available Fe–AC literature does not provide these elements in a sufficiently harmonized form, this section synthesizes published life-cycle evidence and process-level sustainability indicators without representing the analysis as a stand-alone LCA.

Feedstock acquisition is the first life-cycle stage. Agricultural residues such as rice husk, coconut shell, sawdust, and bagasse can provide carbon precursors and may avoid selected waste-management burdens. However, environmental credit for waste use depends on the counterfactual fate of the residue, transport distance, moisture content, preprocessing, and competing uses. These factors should be specified before assigning greenhouse-gas or resource benefits.^50,51^ The synthesis of an Fe_3_O_4_-biochar composite, its application in dye adsorption, and the associated cradle-to-gate life-cycle assessment framework are illustrated in Fig. 5.

**Fig. 5:**
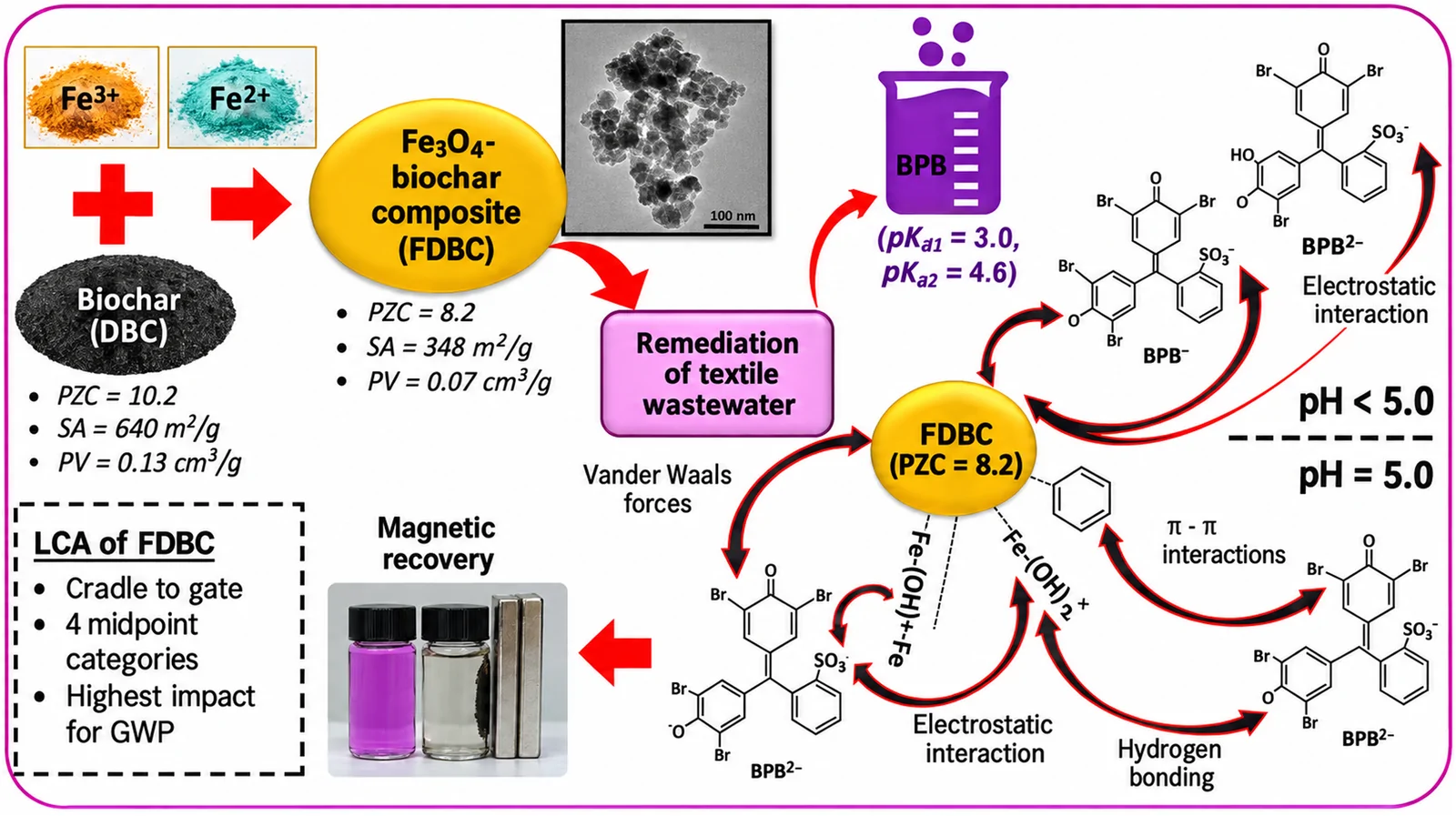
Schematic showing synthesis of an Fe_3_O_4_-biochar composite, its use in dye adsorption, and the associated cradle-to-gate life-cycle assessment framework. Reproduced from ref. 96 with permission from Elsevier, H. Samaraweera *et al.*, “Facile synthesis and life cycle assessment of Iron oxide-Douglas fir biochar hybrid for anionic dye removal from water,” *J. Water Process Eng.*, 2023, **56**, 104377, doi: https://doi.org/10.1016/j.jwpe.2023.104377, copyright 2023. License number: 6067100163310.

Carbonization is a process which involves heating the prepared biomass under an oxygen deficient condition to produce a carbon-rich char. The pore structure is developed during subsequent physical activation with steam or CO_2_ or chemical activation with agents like H_3_PO_4_ and KOH. In some systems a high surface area is achieved at lower peak temperature with chemical activation, but the manufacture, impregnation, washing, recovery, residual salts, and wastewater treatment of the reagents need to be added to the sustainability assessment. Any route that is described as “environmentally preferable” should include a process-specific inventory.^50^

Iron-oxide grafting provides functional groups and, in many formulations, magnetic recoverability; however, it also brings in upstream effects of iron salts, bases, washing water, heating, and drying. Precipitation-based processes can be operated under less severe thermal conditions than hydrothermal processing, but the overall burden will be related to the concentration of reagents, product yield, wastewater generation and chemical recovery. The treatment-normalized material demand is thus more informative than the production burden per kg of sorbent alone.^50,96^

Carbonization, activation, drying and regeneration are significant factors in energy utilization, which is a key parameter of environmental performance. Physical activation can be achieved at high temperatures for long dwell times, while chemical activation can reduce thermal demand and increase chemical and washing requirements. Measured mass and energy balances should be used to evaluate heat recovery, renewable electricity generation, increased activation yield and reagent recycling.^47–50^

The use stage environmental benefit will be dependent on the quantity of compliant water produced and on the mass of contaminant removed from the water, against the quantity of adsorbent, energy, chemical and regeneration used. A high batch removal percentage does not necessarily mean that there is a low environmental burden when the test is performed with a high dose of the adsorbent, a dilute feed or frequent replacement of the material. Treatment normalised metrics are thus essential.^96^

There are three general pathways of regeneration for activated-carbon systems. Thermal regeneration involves the removal or decomposition of the adsorbates by heating, chemical regeneration involves using acids, bases, salts or solvents to desorb or transform the contaminants that are retained, and microbiological regeneration is the degradation of the adsorbates by the use of microorganisms that are compatible with the adsorbates. Process intensification variants are also microwave-assisted thermal treatment and electrochemical methods. The relative sustainability of them is dependent on capacity recovery, energy, reagents, Fe stability, carbon loss and secondary-waste generation.^47–49^ The major life-cycle stages considered for Fe–AC, from agro-waste preparation and carbonization/activation through iron-oxide grafting, water-treatment application, regeneration, and end-of-life management, are summarized in Fig. 6.

**Fig. 6:**
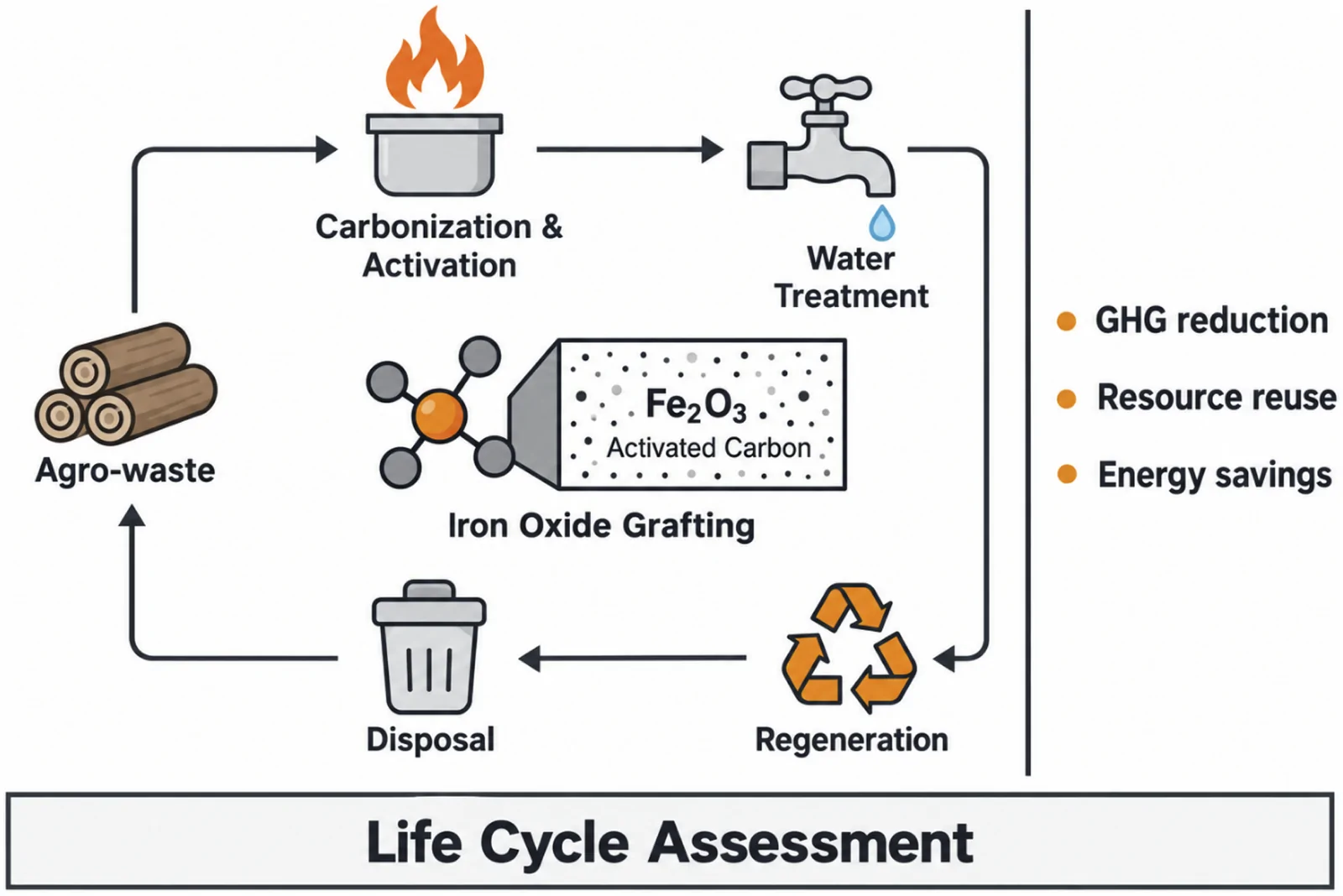
Original synthesis figure summarizing the qualitative Fe–AC life-cycle stages considered in this review: agro-waste preparation, carbonization/activation, iron-oxide grafting, water treatment, regeneration, and end-of-life management.

Repeated regeneration can reduce demand for fresh adsorbent, but environmental benefit depends on the energy and chemicals required per cycle and on the rate of capacity loss. A material that survives more cycles is not necessarily preferable if regeneration causes substantial chemical consumption, iron dissolution, carbon loss, or toxic secondary waste. Regeneration studies should therefore report mass balance, adsorption-capacity retention, Fe release, pore-structure change, and regenerant disposal requirements.^47–49^

The final life-cycle stage is management of spent contaminant-loaded Fe–AC. Even when regeneration extends service life, eventual disposal or material recovery is required. Appropriate options may include contaminant recovery followed by adsorbent reuse, stabilization before regulated disposal, or controlled thermal treatment where emissions and residual ash can be safely managed. Reuse in construction materials or soil applications should not be proposed without leaching and ecotoxicity evidence because the sorbent may contain concentrated hazardous contaminants.^47–50^

Direct environmental comparison between Fe–AC and conventional adsorbents remains uncertain because published studies use different feedstocks, functional units, system boundaries, energy mixes, yields, and assumptions about regeneration and end-of-life. Biomass-derived carbons can reduce selected burdens relative to fossil-derived carbon, but intensive chemical activation, low yield, repeated washing, or energy-demanding drying can offset those gains.^50,96^

Use of agricultural residues can contribute to waste valorization, but it should not automatically be interpreted as carbon sequestration or a carbon-negative process. The net greenhouse-gas balance depends on alternative residue management, process emissions, energy source, product lifetime, transport, and final disposal. These variables should be quantified in future cradle-to-grave assessments.^50,96^

Resource use also depends on activating agents, iron salts, process water, and energy, not only on the carbon precursor. Replacing a fossil-derived precursor with biomass can reduce one resource burden while increasing others if activation yield is low or washing and chemical consumption are high. A multi-indicator assessment is therefore required.^50^

Overall, available evidence supports the potential for agro-waste-derived Fe–AC to reduce selected resource and waste burdens in some scenarios, but the magnitude and direction of benefit remain context dependent. Production energy, activating agents, iron-salt inputs, regeneration, Fe leaching, adsorbent lifetime, and spent-sorbent management can materially influence the result. Sustainability claims should therefore remain conditional until harmonized quantitative assessments with uncertainty and sensitivity analysis are available.^50,96^ The application of Fe_3_O_4_@AC for cationic dye adsorption, followed by magnetic separation, desorption, and adsorbent reuse, is illustrated in Fig. 7.

**Fig. 7:**
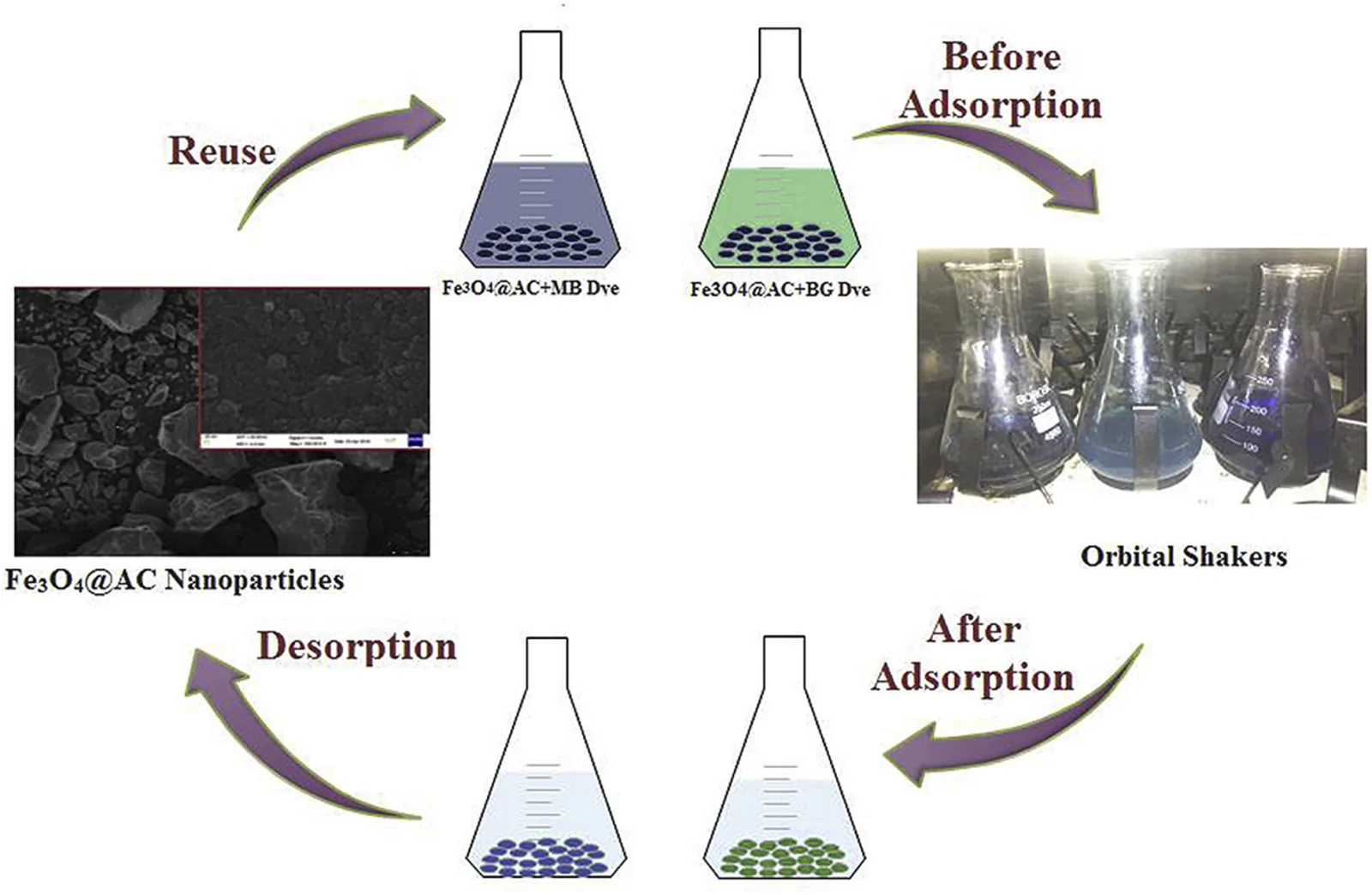
Schematic of Fe_3_O_4_@AC application for adsorption of cationic dyes from water followed by magnetic separation, desorption, and reuse. Reproduced from ref. 98 with permission from Elsevier, S. Joshi, V. K. Garg, N. Kataria and K. Kadirvelu, “Applications of Fe_3_O_4_@AC nanoparticles for dye removal from simulated wastewater,” *Chemosphere*, 2019, **236**, 124280, doi: https://doi.org/10.1016/j.chemosphere.2019.07.011, copyright 2019. License number: 6067100499795.

Table 9 replaces unsupported cross-technology numerical ranges with an evidence-based sustainability summary. Published values for embodied energy, greenhouse-gas emissions, water use, and regeneration are not directly transferable across studies because functional units, boundaries, feedstocks, energy mixes, and treatment objectives differ. Quantitative values are therefore reported only when the cited study defines the relevant experiment or assessment; otherwise the table identifies the evidence needed for a defensible comparison.

**Table 9:** Evidence-based sustainability indicators for Fe–AC and related activated-carbon systems, emphasizing directly supported observations and remaining data gaps

Indicator	Directly supported evidence	Fe–AC interpretation	Required reporting	Current limitation	Useful metric	Ref.
Feedstock/carbon production	Biomass and agricultural residues can replace virgin precursors, but activation conditions strongly affect yield and burden	Potential benefit is feedstock- and process-specific	Moisture, transport, yield, activation agent, temperature, energy source	Counterfactual residue use is often omitted	kg dry precursor and MJ per kg usable adsorbent	50
Iron grafting	Iron salts, base, washing and drying add material and energy inputs	Added burden should be justified by better treatment or recovery	Fe salt and base consumption, Fe retention, wash-water volume, yield	Inventories are rarely treatment-normalized	kg reagents and L wash water per kg composite	52 and 96
Regeneration	Thermal, chemical and microwave-assisted routes can restore capacity but consume energy/reagents	More cycles are beneficial only if Fe and carbon losses remain controlled	Capacity retention, mass loss, Fe release, regenerant/energy per cycle	Few Fe–AC studies provide complete mass balances	% Capacity retained and mg Fe released per cycle	47–49
Iron leaching	Related Fe_3_O_4_-biochar evidence shows strong pH dependence, including no detected Fe at pH 3 but increased release at stronger acidity	Leaching must be evaluated under use and regeneration pH	Dissolved Fe, pH, cycle number, analytical detection limit	Long-duration field monitoring is scarce	mg Fe/L effluent and % Fe inventory lost	81
Real-water performance	Functionalized magnetic carbon has been tested in industrial or pharmaceutical wastewater, but evidence remains limited	Real-water removal can be lower than synthetic-water performance without indicating material failure	DOC/NOM, major ions, turbidity, competing pollutants, flow or EBCT	Few studies combine matrix data, leaching and regeneration	Bed volumes treated; removal to a defined standard	76 and 93
Quantitative LCA	A published iron-oxide/biochar study demonstrates that LCA can be integrated with adsorbent development	Values cannot be transferred directly to different Fe–AC formulations or boundaries	Functional unit, system boundary, inventory, allocation, uncertainty/sensitivity	No harmonized Fe–AC life-cycle dataset	kg CO_2_-eq and energy per m^3^ compliant water	96

A central limitation of the available life-cycle evidence is the absence of harmonized functional units and boundaries. Future studies should follow ISO 14040/14044 principles and use treatment-relevant functional units such as 1 m^3^ of water treated to a defined effluent standard or a defined mass of contaminant removed from a specified matrix. Sensitivity analysis should include carbonization energy, activating-agent recovery, iron-salt production, adsorbent yield and lifetime, regeneration, Fe leaching, and end-of-life scenarios. Without this harmonization, numerical carbon-footprint comparisons remain indicative rather than definitive.^96^

## 6 Potential for sustainable and large-scale application

Fe–AC has potential for larger-scale water treatment because biomass precursors can be widely available and precipitation-based iron grafting can be implemented using comparatively simple unit operations. Practical suitability, however, depends on feedstock standardization, composite mechanical strength, hydraulic performance, iron stability, regeneration, and compliance with treated-water and waste-disposal requirements.^63,68^

Agricultural residues can provide a distributed precursor base, but variability in ash content, lignocellulosic composition, moisture, and local availability affects product reproducibility. Scale-up of co-precipitation also requires uniform mixing, controlled nucleation, reproducible Fe loading, efficient washing, and management of salt-containing process water. These factors should be demonstrated at kilogram and pilot scales before industrial feasibility is inferred.^63,65,68^

A realistic evaluation of scalability requires evidence from continuous-flow systems and real waters. Competing cations and anions can suppress adsorption by occupying charged or coordinative sites, while natural organic matter can block pores, complex dissolved metals, alter surface charge, and foul the composite. Iron leaching is especially important under acidic or strongly complexing conditions because loss of the Fe phase can reduce performance and introduce secondary contamination. Continuous columns should therefore report breakthrough curves, bed volumes treated, pressure drop, Fe in the effluent, regeneration performance, and changes in capacity over repeated cycles.

## 7 Challenges and future perspectives

### 7.1 Current limitations and challenges in synthesis, characterization, and application

Fe–AC synthesis and application face several interconnected challenges. Non-uniform nucleation can produce agglomerated iron-oxide domains and heterogeneous active-site distributions, reducing accessible surface area and causing batch-to-batch variability. Hydrothermal and sol–gel methods can improve structural control but usually require tighter process control and, in many cases, greater energy or equipment input.^63^

Advanced characterization methods such as TEM and XPS provide valuable information on nanoparticle dispersion and surface chemical state, but they require specialized instrumentation and expertise. Routine quality control can therefore combine accessible screening methods such as FTIR, XRD, BET analysis, elemental analysis, and VSM with periodic high-resolution characterization. Proxy methods should be validated against the property they are intended to represent rather than used as substitutes without calibration. The overall evidence pathway linking Fe–AC precursor selection and synthesis to material structure, contaminant-specific adsorption mechanisms, real-water constraints, engineering validation, and sustainability assessment is summarized in Fig. 8.

**Fig. 8:**
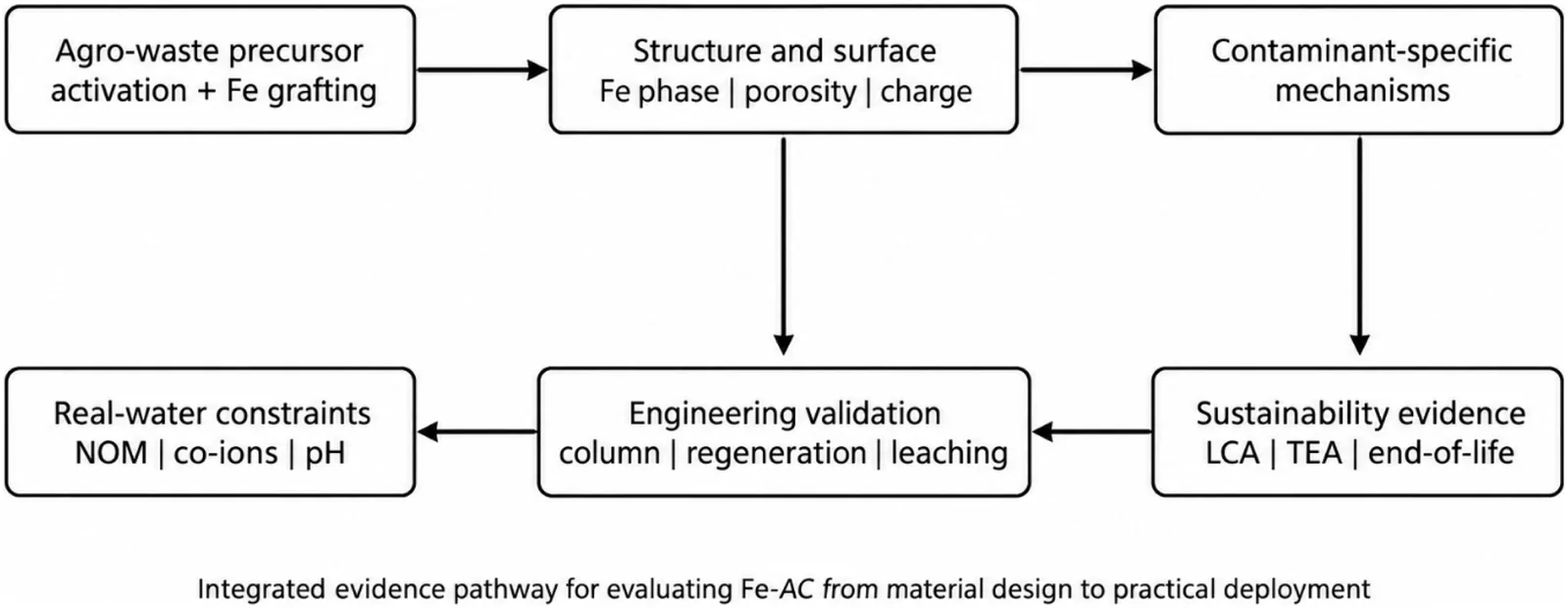
Original integrative evidence pathway linking Fe–AC precursor selection and synthesis with structure, contaminant-specific mechanisms, real-water constraints, engineering validation, and sustainability assessment.

During application, fouling and irreversible binding can reduce capacity over repeated cycles. Natural organic matter and co-existing ions can compete for adsorption sites, alter surface charge, or complex target metals. Acidic regeneration can dissolve iron phases, while aggressive thermal treatment can change pore structure and surface functionality. Spent Fe–AC containing hazardous contaminants requires leaching assessment and controlled end-of-life management. The quantitative pH-dependent Fe-release evidence discussed in Section 3.7 illustrates why leaching should be treated as a performance metric rather than only as a qualitative risk.^81^

The principal challenges in Fe–AC synthesis, characterization, and application are mapped to mitigation strategies and their current evidence status in Table 10.

**Table 10:** Systematic mapping of key challenges in Fe–AC composite synthesis, characterization, and water-treatment application to proposed mitigation strategies. NOM = natural organic matter; NPs = nanoparticles

Challenge	Root cause	Proposed mitigation strategy	Evidence status	Priority
Non-homogeneous distribution of Fe NPs on AC surface	Uncontrolled nucleation during co-precipitation; insufficient AC surface functionalisation	Pre-functionalise AC with carboxyl/hydroxyl groups; use sonochemical or microwave-assisted grafting; controlled pH and temperature during co-precipitation	Lab-scale demonstrated; pilot-scale not yet reported	High
Loss of adsorption capacity upon repeated regeneration	Blockage of active sites by irreversibly bound pollutants; partial dissolution of Fe oxide phase during acid/alkali regeneration	Optimize regeneration reagent concentration; explore photo-Fenton or electrochemical regeneration; develop core–shell Fe@AC architectures	Partial lab-scale evidence; no long-term (more than 10 cycle) pilot data	High
Iron leaching into treated water	Dissolution of surface Fe under acidic conditions; incomplete surface coating	Stabilize iron attachment; avoid unnecessarily aggressive acidic operation/regeneration; measure dissolved Fe during use and cycling	Quantitative pH-dependent Fe-release evidence exists for a related Fe_3_O_4_-biochar system,^81^ but long-duration Fe–AC field data remain scarce	High
Scalability of hydrothermal and sol–gel synthesis	Energy-intensive conditions (high pressure/temperature); batch-scale limitation	Transition to continuous-flow reactor designs; co-precipitation under ambient conditions as scalable alternative	Laboratory and some kilogram-scale preparation evidence exists; continuous production and long-duration Fe–AC column validation remain limited	High
Selectivity in multi-contaminant real wastewater	Competing ions and NOM reduce target pollutant uptake; interference from humic substances	Surface modification with selective chelating ligands (aminopropyl, thiol groups); pH-controlled adsorption protocols; NOM pre-filtration	Selected real-wastewater and complex-matrix studies are available,^76,84,93^ but systematic pilot-scale datasets remain limited	Critical
High cost and complexity of advanced characterization	TEM and XPS require specialized equipment and expertise; limited access in low-resource settings	Use tiered quality control: routine FTIR/XRD/BET/VSM/elemental and leaching tests, validated periodically by TEM/XPS or equivalent advanced methods	Individual methods are established; a standardized Fe–AC proxy protocol has not yet been validated	Moderate
Regulatory framework for Fe–AC nanocomposite disposal	Fe NPs classified as potential nanomaterial hazard in several jurisdictions; no harmonized disposal standard	Develop leachability and ecotoxicological profiles of spent Fe–AC; engage regulatory bodies for nanomaterial classification	Research-stage evidence; spent-sorbent classification and disposal requirements are jurisdiction-specific	Moderate

### 7.2 Potential for improvement and innovation

Improvement of Fe–AC performance requires controlled iron loading and dispersion without excessive pore blockage. Surface pre-functionalization, staged precipitation, sonochemical mixing, and optimized pH can improve nucleation and attachment, but any gain should be evaluated together with Fe stability, reagent use, yield, and batch-to-batch reproducibility.^63^

Routine characterization would benefit from validated, lower-cost screening protocols. FTIR, XRD, VSM, elemental analysis, and simple leaching tests can support production quality control, while TEM, XPS, and advanced spectroscopy can be reserved for mechanistic verification and periodic validation. The objective should be a tiered characterization strategy with known uncertainty rather than a claim that inexpensive methods provide equivalent information.

Longer adsorbent life can improve practical and environmental performance only when regeneration preserves both carbon porosity and the iron phase. Future studies should combine capacity measurements with Fe leaching, adsorbent mass loss, BET surface-area change, surface-speciation analysis, contaminant recovery or destruction, and regenerant consumption. Reporting removal percentage after only a few cycles is insufficient to establish long-term durability.^47–49^

## 8 Future research directions

### 8.1 Enhancing adsorption capacity and selectivity

Future studies should optimize iron loading, nanoparticle dispersion, pore accessibility, and surface functional groups while maintaining mechanical and chemical stability. Selectivity should be evaluated in multicomponent matrices rather than inferred from single-solute tests. Mechanistic studies that combine spectroscopy, contaminant speciation, and computational modeling can help distinguish electrostatic attraction, inner-sphere complexation, ligand exchange, precipitation, redox transformation, hydrogen bonding, and π–π interactions.

### 8.2 Creating less hazardous synthesis protocols

Greener synthesis should be assessed using measurable criteria such as reagent hazard, process yield, E-factor, water consumption, energy demand, washing burden, and Fe stability rather than by the use of a plant extract alone. Low-temperature precipitation, reagent recovery, aqueous processing, and renewable-energy integration are promising only when they reduce the complete process burden.

### 8.3 Scaling up

Industrial translation requires synthesis routes that maintain uniform Fe loading, pore accessibility, magnetic response, particle strength, and low leaching at larger batch sizes. Continuous or semi-continuous production should be coupled with column testing, hydraulic evaluation, regeneration, and effluent monitoring so that material performance is assessed under treatment-relevant conditions.

Pilot-scale and field studies are required to establish performance in real wastewater and under realistic flow conditions. Collaboration among materials researchers, water-treatment engineers, analytical laboratories, and regulators is needed to define treated-water targets, particle containment, spent-sorbent classification, regeneration controls, and safe disposal pathways.

### 8.4 Advancing green and bio-inspired synthesis

Plant-derived polyphenols and other biogenic reagents can assist iron-oxide formation or stabilization, but their environmental advantage should be verified against conventional routes using yield, reagent toxicity, water demand, energy use, reproducibility, and Fe leaching. A one-pot route is valuable only when it provides a measurable reduction in total process burden.

### 8.5 Developing multifunctional Fe–AC systems

Magnetic recovery, selective functional groups, and combined adsorption-redox functions may expand Fe–AC treatment of pesticides, pharmaceuticals, and other emerging contaminants. Future designs should prioritize selectivity in realistic matrices, controlled particle release, regeneration, and compatibility with continuous-flow contactors rather than adding functions that are demonstrated only in idealized batch tests.

The main research gaps, their scientific rationale, proposed experimental or analytical approaches, and measurable success criteria are summarized in Table 11.

**Table 11:** Research-gap matrix for iron oxide-grafted activated carbon composites in water treatment. DFT = density functional theory; LCA = life-cycle assessment; TEA = techno-economic analysis; CAPEX = capital expenditure; OPEX = operational expenditure; GAC = granular activated carbon

Research gap	Scientific rationale	Suggested approach	Success criterion/reporting target
Standardized performance benchmarking across studies	Current adsorption data cannot be meaningfully compared because initial concentration, pH, dose, and temperature vary widely across studies	Use a proposed harmonized benchmark matrix with explicitly reported pH, *C*_0_, adsorbent dose, temperature, ionic strength, contact time and analytical method; report both *q* and removal percentage	Sufficient multi-laboratory adoption to permit direct comparison and uncertainty analysis
Long-term regeneration performance (more than 20 cycles)	Most studies report only a small number of cycles; longer cycling is required to quantify capacity, mass and Fe loss	Run extended adsorption–regeneration cycles; measure *q*_max_ or working capacity, Fe release, mass loss and BET/pore changes at defined intervals	Pre-defined capacity-retention and Fe-leaching targets appropriate to the intended water-treatment application
Pilot-scale and real-wastewater validation	Most Fe–AC evidence remains laboratory and batch based; available real-water studies are important but not yet sufficient for broad engineering generalization	Conduct continuous-flow column studies with real municipal or industrial effluent; report influent matrix, EBCT/flow, breakthrough curves, bed volumes, pressure drop, Fe release and regeneration	Stable treatment to a defined effluent criterion over a treatment-relevant number of bed volumes
Mechanistic differentiation across contaminant classes	Quantitative evidence distinguishing contributions of inner-sphere complexation, electrostatic attraction, and pi–pi interaction under environmentally relevant conditions is lacking	Combine *in situ* FTIR/ATR, XPS, and DFT modeling to quantify bond energies and surface speciation for each contaminant class	DFT-confirmed mechanism for at least three contaminant classes with experimentally validated surface speciation data
Greener synthesis routes and green chemistry metrics	Fe–AC synthesis currently uses strong acids, alkalis, or high-temperature conditions; green chemistry metrics (E-factor, atom economy) are not reported	Compare candidate green routes against a conventional co-precipitation baseline using yield, energy, water demand, reagent hazard, E-factor and Fe stability	Lower measured process burden without loss of treatment performance or stability
Quantitative LCA with harmonized functional units	Fe–AC life-cycle studies use different boundaries and functional units, preventing robust numerical comparison	Conduct cradle-to-grave assessment following ISO 14040/14044 with a treatment-based functional unit and explicit regeneration/end-of-life scenarios	Peer-reviewed assessment with transparent inventory, uncertainty and sensitivity analysis
Techno-economic analysis at industrial scale	Cost estimates in existing literature cover only material production; OPEX, maintenance, and waste disposal are not included	Develop TEA covering CAPEX, OPEX, adsorbent replacement/regeneration, analysis, pumping and residuals disposal at a defined plant capacity	Treatment cost reported per m^3^ of water meeting a defined quality target, with sensitivity analysis

## 9 Conclusions

Agro-waste-derived Fe–AC composites combine the porous adsorption capacity of activated carbon with iron-oxide surface chemistry and, in many formulations, magnetic recoverability. The evidence supports multiple mechanisms rather than a single universal pathway: inorganic ions can undergo inner-sphere complexation, ligand exchange, ion exchange, precipitation, electrostatic attraction, and redox transformation, whereas organic contaminants are governed more strongly by pore filling, hydrophobic interactions, pi–pi stacking, hydrogen bonding, electrostatic effects, and selected Fe–O coordination. Synthesis route and iron loading influence crystallinity, dispersion, pore accessibility, and stability, making structure–property relationships central to performance.

The strongest evidence for Fe–AC remains at laboratory scale. Reported adsorption capacities demonstrate material potential but cannot be directly ranked across studies without accounting for pH, initial concentration, adsorbent dose, temperature, contact time, water matrix, and test configuration. Practical translation is constrained by natural organic matter, competing ions, iron leaching, regeneration losses, particle handling, and the limited availability of continuous-flow, pilot-scale, and real-wastewater data. Environmental advantages associated with agro-waste feedstocks and regeneration are plausible but remain dependent on energy, reagent, transport, lifetime, and end-of-life assumptions.

Future development should prioritize standardized benchmarking, molecular-level mechanistic validation, long-term regeneration with iron-leaching control, continuous-flow studies, and testing in complex real waters. Quantitative life-cycle and techno-economic assessments should use harmonized treatment-based functional units and explicitly include production, regeneration, secondary waste, and end-of-life scenarios. Progress in these areas will determine whether Fe–AC can move from promising laboratory adsorbents to reliable and environmentally responsible water-treatment materials.

## Author contributions

Bijoylakshmi Boruah: conceptualization, literature review, data collection, systematic analysis, manuscript preparation, and writing. Dr Pratap Kumar Swain: supervision, critical review of methodology, interpretation of findings, and revision of manuscript. Dr Manash Ranjan Das: co-supervision, critical appraisal of content, guidance on technical aspects, and manuscript revision.

## Conflicts of interest

The authors declare no competing financial or non-financial interests.

## Data Availability

No primary research results, software or code have been included and no new data were generated or analysed as part of this review.
